# Global trends in grassland carrying capacity and relative stocking density of livestock

**DOI:** 10.1111/gcb.16174

**Published:** 2022-04-06

**Authors:** Johannes Piipponen, Mika Jalava, Jan de Leeuw, Afag Rizayeva, Cecile Godde, Gabriel Cramer, Mario Herrero, Matti Kummu

**Affiliations:** ^1^ 174277 Water and Development Research Group Aalto University Espoo Finland; ^2^ 5228 Department of Bioecology Baku State University Baku Azerbaijan; ^3^ 5228 SILVIS Lab Department of Forest and Wildlife Ecology University of Wisconsin‐Madison Madison Wisconsin USA; ^4^ Commonwealth Scientific and Industrial Research Organisation, Agriculture and Food St Lucia QLD Australia; ^5^ Department of Global Development College of Agriculture and Life Sciences and Cornell Atkinson Center for Sustainability Cornell University Ithaca New York USA

**Keywords:** aboveground biomass, feed, grasslands, interannual variability, MODIS, net primary production, overgrazing, rangelands

## Abstract

Although the role of livestock in future food systems is debated, animal proteins are unlikely to completely disappear from our diet. Grasslands are a key source of primary productivity for livestock, and feed‐food competition is often limited on such land. Previous research on the potential for sustainable grazing has focused on restricted geographical areas or does not consider inter‐annual changes in grazing opportunities. Here, we developed a robust method to estimate trends and interannual variability (IV) in global livestock carrying capacity (number of grazing animals a piece of land can support) over 2001–2015, as well as relative stocking density (the reported livestock distribution relative to the estimated carrying capacity [CC]) in 2010. We first estimated the aboveground biomass that is available for grazers on global grasslands based on the MODIS Net Primary Production product. This was then used to calculate livestock carrying capacities using slopes, forest cover, and animal forage requirements as restrictions. We found that globally, CC decreased on 27% of total grasslands area, mostly in Europe and southeastern Brazil, while it increased on 15% of grasslands, particularly in Sudano‐Sahel and some parts of South America. In 2010, livestock forage requirements exceeded forage availability in northwestern Europe, and southern and eastern Asia. Although our findings imply some opportunities to increase grazing pressures in cold regions, Central Africa, and Australia, the high IV or low biomass supply might prevent considerable increases in stocking densities. The approach and derived open access data sets can feed into global food system modelling, support conservation efforts to reduce land degradation associated with overgrazing, and help identify undergrazed areas for targeted sustainable intensification efforts or rewilding purposes.

## Graphical abstract

We estimated aboveground biomass (AGB) and carrying capacity (CC) over 2001–2015 based on remote sensing and compared those estimates with gridded livestock populations. We found strong negative trends in CC in Europe and southeastern Brazil and potential overgrazing in northwestern Europe, and southern and eastern Asia.

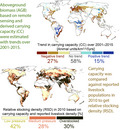

## 1 INTRODUCTION

Opinions on the sustainability of livestock production on global grasslands vary across the scientific literature. Several studies suggest that some of the livestock production relying on natural grasslands is environmentally sustainable (e.g., Holechek et al., 2010; Kemp & Michalk, 2007) and that considerable parts of global grasslands are understocked, thus providing the potential to increase the production of animal proteins in these areas (Fetzel, Havlik, Herrero, & Erb, 2017; Monteiro et al., 2020; Rolinski et al., 2018). However, other studies postulate that a notable fraction of the world's grasslands hosts livestock populations that exceed the carrying capacity (CC), with negative impacts on the environment (Alkemade et al., 2013; Reid et al., 2009; Wolf et al., 2021). Yet, these contrasting views do not necessarily contradict each other. Instead, they can reflect a situation where some grasslands are overstocked, while others are used according to, or below, their CC. However, some studies explicitly disagree; for example, Irisarri et al. (2017) argue that grazing intensities reported by Fetzel, Havlik, Herrero, & Erb (2017) are underestimated. This all indicates the complexity of grazing‐related global research, and shows that we still lack information regarding grazing opportunities, threats and the best measurement methods related to stocking densities.

Depending on the literature source, grasslands comprise 20%–47% of the world's land area (Arneth et al., 2019; Godde et al., 2018). Furthermore, they support the livelihoods of around 800 million people (Gibson & Newman, 2019; Kemp et al., 2013; Suttie et al., 2005). Grazing systems are diverse, ranging from nomadic pastoral activities in sub‐Saharan native savannas to sedentary Dutch dairy farming on fertilized sown pastures (Godde et al., 2018). In some regions, vegetation adapted to extreme conditions and the species‐rich population of the grasslands provide a buffer for the disadvantageous effects of climate change (Craine et al., 2013; Dengler et al., 2014; Tamburino et al., 2020). In fact, constitutive components of biodiversity such as pollinators are greatly dependent on these regions (Klaus et al., 2021). However, moving away from traditional agricultural practices and towards intensive grazing jeopardizes grassland areas and their indigenous species (Estel et al., 2018; Gibson & Newman, 2019; Gossner et al., 2016).

Heavy stocking densities and overgrazing may cause land degradation and desertification, leading to land erosion, whereas properly managed grazing can contribute to the provision of ecosystem services (Bengtsson et al., 2019), regulating the terrestrial carbon cycle and increasing the ecological resilience against natural disasters (Gibson & Newman, 2019; Lv et al., 2020; Wang & Tang, 2019). The importance of avoiding heavy stocking pressure and the benefits of rotational grazing has been extensively emphasized in the literature (Filazzola et al., 2020; Gibson & Newman, 2019; Holechek et al., 2010; Loeser et al., 2007; Wang & Tang, 2019). The biodiversity effects of different grazing pressures vary along environmental gradients and trophic levels (Filazzola et al., 2020; Wang & Tang, 2019) making generalizations difficult. According to a meta‐analysis of Wang and Tang (2019), grazing enhances biodiversity by increasing plant and microbial diversity but weakens it by decreasing arthropod diversity. Another meta‐analysis (Filazzola et al., 2020) implies that exclusion of livestock increases plant abundance, but the relationship between plant diversity and grazing is more mixed. Moreover, livestock exclusion increases the abundance and diversity of all invertebrates and vertebrates apart from detritivores (Filazzola et al., 2020). Regions with extremes in temperature or precipitation are most sensitive to grazing (Filazzola et al., 2020); thus, management of biodiversity in these regions is particularly critical. Due to these complex interactions, the exact locations where grazing should increase or decrease to improve environmental sustainability and biodiversity would still need further studies.

As highlighted in the literature, CC is important for determining proper stocking rates, as it describes the maximum number of animals or animal units an area can sustainably hold (De Leeuw et al., 2019; Rees, 1996). Although the principles of the CC calculations are straightforward, evaluation of the available forage creates difficulties due to the year‐to‐year variation of grass yields and the local geographical restrictions such as tree cover and terrain slope. So far, CC assessments based on remote sensing have mainly been applied to restricted geographical areas (e.g., De Leeuw et al., 2019 in the mountain grasslands of Azerbaijan; Zhao et al., 2014 in the Xilingol grassland of Northern China). Only a few studies have globally estimated potential grazing intensities either based on MODIS net primary productivity (NPP) data (Petz et al., 2014) or biophysical models (Fetzel, Havlik, Herrero, & Erb, 2017; Monteiro et al., 2020; Rolinski et al., 2018) or a combination of both (Fetzel, Havlik, Herrero, Kaplan, et al., 2017; Wolf et al., 2021). However, these studies include limited temporal coverage (apart from Wolf et al., 2021) and are therefore unable to observe changes over a longer timeframe, thus lacking the ability to capture the year‐to‐year dynamics impacting the CC. In addition, assumptions related to the aboveground fraction of NPP are only valid for individual ecosystems (Fetzel, Havlik, Herrero, & Erb, 2017; Fetzel, Havlik, Herrero, Kaplan, et al., 2017; Petz et al., 2014; Wolf et al., 2021), or the resolution used in the abovementioned global studies is relatively coarse (30 arc‐min in Fetzel, Havlik, Herrero, & Erb, 2017; Rolinski et al., 2018; Wolf et al., 2021). Therefore, there is a need for robust observation‐based (i.e., remote sensing) estimates for CC, spanning multiple years with a high spatial resolution.

The aim of this study is to estimate changes in CC by calculating yearly CC values between 2001 and 2015 based on remotely sensed aboveground biomass (AGB). This allows us to assess the trend and interannual variability (IV) in CC, missing from existing literature. Additionally, we aim to assess the stocking density relative to the primary production that is available to sustain livestock. Global assessment of the stocking density of livestock exists (Gilbert et al., 2018), but these estimates cannot directly express which areas are overstocked or understocked. The stocking density that can be sustained depends on the availability of forage biomass, which varies geographically. Here we calculate the relative stocking density (RSD)—that is, the ratio of stocking density relative to the availability of forage biomass—to estimate grass‐biomass utilization in 2010. This is done for all grasslands as well as only for “livestock‐grazing” lands where livestock mainly relies on grass biomass. On top of these new findings, we also improved the methodology for estimating the AGB and CC from MODIS NPP on various fronts. First, we used temperature as a predictor when allocating a fraction of total NPP to AGB, instead of a single constant used in existing global grazing studies (Fetzel, Havlik, Herrero, & Erb, 2017; Fetzel, Havlik, Herrero, Kaplan, et al., 2017; Petz et al., 2014; Wolf et al., 2021). Second, we developed a relationship between forest cover and available biomass, based on a meta‐analysis, to estimate the AGB. This approach contrasts with existing methods that either exclude woody areas from the analysis (Petz et al., 2014; Wolf et al., 2021) or use a single reduction factor for all areas that include trees (Fetzel, Havlik, Herrero, & Erb, 2017). Finally, we included a detailed uncertainty assessment in our analysis to capture some of the uncertainties associated with our estimates in the different world regions.

We expect the results to reveal two types of areas: grasslands where CC values fluctuate significantly over the years, and areas where they remain more stable. Moreover, we aim to identify grasslands that can be particularly at risk of overgrazing in the absence of adequate supplemental feeding, and grasslands where grazing intensity falls within the CC as well as the trend over time in the CC levels. In addition, we analyze factors that might prevent the currently estimated animal densities to reach the theoretical CC boundaries and discuss why transgressing these upper boundaries is inadvisable. Detected changes in CC and RSD help us to anticipate the future and prevent undesirable environmental impacts of livestock grazing.

## 2 MATERIALS AND METHODS

### 2.1 Net primary productivity

**FIGURE 1:**
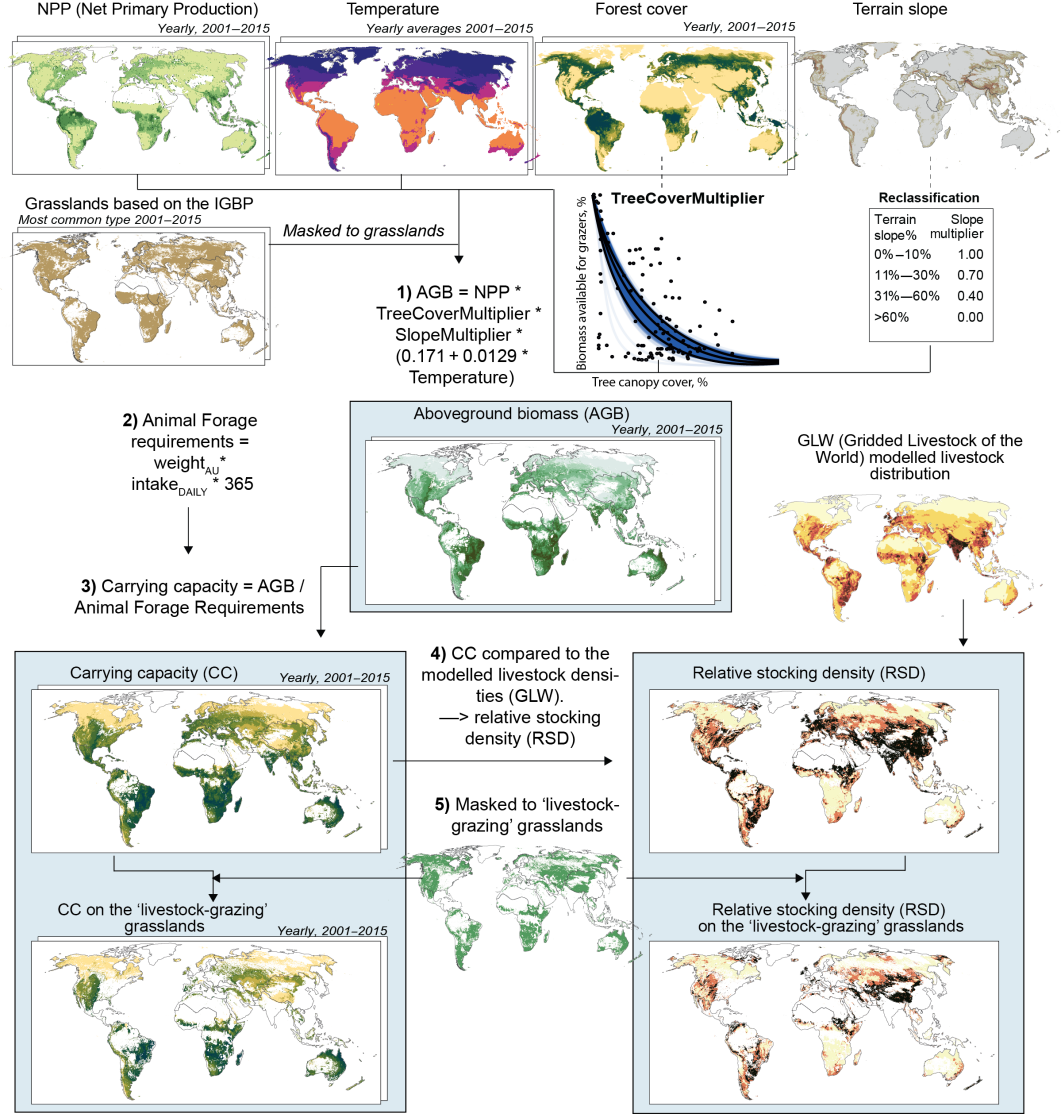
Flowchart of data and methods used in the analysis. Abbreviations used in the study: AGB, aboveground biomass; AU, animal unit; CC, carrying capacity; GLW, gridded livestock of the world; IGBP, the International Geosphere‐Biosphere Programme; NPP, net primary productivity; RSD, relative stocking density.

To conduct the analysis, we combined several open‐access global data sets together as summarized in Figure 1. A detailed description is given below. We estimated CC (i.e., CC) using MODIS (Moderate Resolution Imaging Spectroradiometer) data products. We first extracted MODIS Land Cover Type (Sulla‐Menashe & Friedl, 2018; Table 1) and chose classes with significant grass cover—that is, Woody savannas, Savannas and Grasslands according to the IGBP (International Geosphere–Biosphere Programme) classification system. This grassland area comprises around 43% of the world's land area (excluding Antarctica). As land cover types might vary between years, we calculated the mode value (the land cover class that occurs most often) over our study period of 2001–2015. The study period was restricted by data availability (see below).

**TABLE 1:** Data used in the analysis

Data	Time interval	Resolution	Reference
Land cover type (MCD12Q1.006)	Mode value of 2001–2015	500 m (16.2 arc‐seconds)	Sulla‐Menashe and Friedl (2018)
Net primary productivity (MOD17A3HGF)	Yearly averages for 2001–2015	500 m (16.2 arc‐seconds)	Running and Zhao (2019)
Mean annual temperature (TerraClim)	Yearly averages for 2001–2015	4 km (2.5 arc‐ minutes)	Abatzoglou et al. (2018)
Global 30 m lands at tree canopy version 4	Yearly averages for 2001–2015	30 m (1 arc‐seconds)	Sexton et al. (2013)
Terrain slope% map	—	250 m (7.5 arc‐seconds)	Amatulli et al. (2020)
Gridded livestock of the world (GLW 3)	Year 2010	10 km (5 arc‐minutes)	Gilbert et al. (2018)
Global livestock production systems	Year 2011	1 km (30 arc‐seconds)	Robinson et al. (2011)

Second, we followed the approach described by De Leeuw et al. (2019) to calculate AGB based on the 500 m resolution MODIS NPP (Running & Zhao, 2019; Table 1). We calculated the yearly NPP values during 2001–2015 and used a carbon conversion factor (Eggleston et al., 2006; Table 2) to convert the original NPP values expressed as carbon per unit area to g m^− 2^ year^−1^ biomass. Because plants store part of their NPP in belowground biomass, we used the following formula (Equation 1) developed for the grasslands (Hui & Jackson, 2006) to derive the fraction of the NPP (*f*_ANPP_) allocated AGB:

(1) $$f_{\text{ANPP}}=0.171+0.0129\;\text{MAT}\;\left(\text{Hui}\;\&\;\text{Jackson,}\;2006\right),$$

where MAT is the mean annual temperature in °C. Yearly MAT values for 2001–2015 were derived from TerraClimate–Climatology Lab (Abatzoglou et al., 2018) and resampled to 500 m resolution (Table 1).

**TABLE 2:** Overview of uncertainty estimates used in the analysis. See the justifications for the limits in Appendix S2

Variable	Distribution	*n*	Lower limit	Upper limit	Mean	SD
Forage requirement	Truncated normal distribution	1000	0.018	0.04	0.02	—
Carbon conversion factor	Truncated normal distribution	1000	0.47	0.50	—	—
Conversion factors: cattle	Truncated normal distribution	1000	0.50	1.25	1.00	—
Conversion factors: buffalo	Truncated normal distribution	1000	0.60	0.70	—	—
Conversion factors: sheep	Truncated normal distribution	1000	0.10	0.15	—	—
Conversion factors: goat	Truncated normal distribution	1000	0.10	0.15	—	—
Conversion factors: horse	Truncated normal distribution	1000	0.40	1.80	—	—
*TreeCoverMultiplier*	Truncated normal distribution	1000	Bottom 2.5% curve	Top 97.5% curve	Median curve	—
NPP	Uniform distribution	1000	—	—	1	0.07
*f*ANPP	Uniform distribution	1000	—	—	1	0.198 × 0.71

We chose to use MODIS‐derived NPP product instead of modelled NPP products due to its much higher resolution (16.2 arc‐seconds or 500 m in the equator compared with 30 arc‐minutes or 50 km in the equator), which allowed us to use high‐resolution data for other input variables (see Table 1) and thus more accurate estimates for AGB and other assessed variables. Moreover, we wanted to use data that are based on observations (here remote sensing), and continuously updated. Nevertheless, to explore the robustness of our MODIS derived AGB estimates, we also calculated AGB using simulated NPP data from the Inter‐Sectoral Impact Model Intercomparison Project Phase 2a (ISIMIP2a, see Reyer et al., 2019). Differences between modelled and observed AGB estimates and uncertainties are discussed later in this article as well as in the Appendix (S2.10 and Figure S9 in the Appendix).

### 2.2 Aboveground biomass

Trees in savannas and woody savannas compete with grass and reduce its productivity. We reviewed the literature related to the effect of tree canopy cover on the ground cover and the NPP of sub‐canopy vegetation (De Leeuw & Tothill, 1990; Le Brocque et al., 2008; Lloyd et al., 2008; White et al., 2000). These studies revealed that an increase in the tree canopy cover results in a non‐linear reduction in the sub‐canopy cover that is available to grazers. Based on this, and especially on scatter plots provided by Le Brocque et al. (2008) and Lloyd et al. (2008), we fitted the following transfer function (see Appendix S2.4) to translate the tree canopy cover into the fraction of NPP that is allocated to the sub‐canopy and available for grazers. We assumed that the derived sub‐canopy vegetation (Equation 2) also includes consumable leaves and shrubs while acknowledging that this might not be the case in all the ecosystems (see Appendix S2.4). Nevertheless, this captures the available biomass in the majority of the grasslands and although there are still uncertainties, it is a considerable improvement to the currently used methods which either do not include woody areas in the analysis (Petz et al., 2014; Wolf et al., 2021) or use a single reduction factor despite the canopy cover density (Fetzel, Havlik, Herrero, & Erb, 2017).

(2) $$\begin{array}{c} TreeCoverMultiplier=\frac{1}{e^{4.45521\;\times \;x}},\;\;\;\;\;\;x\in \left\{0,1\right\},TreeCoverMultiplier\in \left\{1,0\right\}, \end{array}$$

where *TreeCoverMultiplier* refers to sub‐canopy biomass and *x* refers to the fraction of the pixel area covered by the tree canopy. Here, we used Global 30 m Landsat Tree Canopy data provided by Sexton et al. (2013; Table1) due to its high local accuracy, precision and suitability for application over arbitrary time periods (Sexton et al., 2015, 2016). Sexton et al. (2013) cover the years 2000, 2005, 2010, and 2015 but do not include data for each year, so we interpolated the data to include all years 2001–2015. Based on the *TreeCoverMultiplier* function (Equation. 2; Figure S1 in the Appendix), we reclassified the original values and derived the AGB of the understory (Figure 1). After the reclassification, we resampled the data to the MODIS resolution of 500 m. Thus, the final modified forest coverage map expresses the feed efficiency number for each pixel. The specific procedure for creating the *TreeCoverMultiplier* function can be found in Appendix S2.4.

We further reduced AGB by a terrain slope steepness factor (see De Leeuw et al., 2019) to account for the risk of erosion and avoid land degradation (Holechek et al., 2010). Data for the global representation of terrain slope steepness at 250 m resolution is available from Amatulli et al. (2020; Table 1). We first reclassified the terrain slopes following the recommendations of George and Lyle (2009) and then resampled the data to the same resolution as the MODIS products. Thus, the modified terrain slope map (*SlopesMultiplier* in Equation 3) expresses the feed efficiency number for each pixel.

Given the above, the formula for AGB available for grazing animals (biomass g m^− 2^ year^−1^) in a year *i* is (Equation 3):

(3) $${\text{AGB}}_{i}=\frac{{\text{NPP}}_{i}\times f{\text{ANPP}}_{i}}{\text{carbon}\;\text{conversion}\;\text{factor}}\times TreeCoverMultiplier_{i}\times SlopesMultiplier,$$

### 2.3 Carrying capacity and relative stocking density

After calculating AGB, we estimated the CC in animal units (AU) per unit area per year. Following the definition of Holechek et al. (2010), the AU corresponds to 455 kg, with a daily forage intake varying between 1.8% and 4% dry matter of its body weight (Table 2; see S1 in the Appendix). We then aggregated the daily dry matter intake for a year. The available AGB divided by the forage requirements of the AU yields the CC (Equation 4):

(4) $${\text{CC}}_{i}=\frac{{\text{AGB}}_{i}}{{\text{weight}}_{\text{AU}}\times {\text{intake}}_{\text{daily}}\times 365},$$

where AGB*_i_* is derived from Equation 3 (converted to kg biomass km^−2^ year^−1^), weight equals to 455 kg/AU and daily intake (unitless fraction) ranges from 0.018 to 0.04, resulting in CC_i_ in AU km^–2^ year^–1^. As a final step, we derived the RSD by dividing the modelled livestock density by the potential density that could be sustained while considering grass biomass availability alone. This calculation creates a ratio that varies from zero to above one. (Equation 5):

(5) $$\text{Relative}\;\text{stocking}\;{\text{density}}_{i}=\frac{\text{Animal}\;\text{units}}{\text{CC}}.$$

First, we extracted the Gridded Livestock of the World (GLW 3) estimates for the year 2010 (Gilbert et al., 2018; Table 1) and converted the number of cattle, horses, sheep, goats, and buffaloes to the number of animal units per unit area, following FAO (2011) and Holechek et al. (2010). We used the dasymetric GLW product in the analysis, but also tested using the other available product, that is, areal‐weighted one, which resulted in similar RSD estimates (see Figure S4 in the Appendix).

We classified the RSD into three classes based on the literature (see below): <0.20 low pressure, 0.20–0.65 medium pressure, >0.65 overstocked. These class boundaries were used to allow for factors that prevent livestock from consuming all AGB; part of it is trampled, consumed by other species, or avoided because of toxicity or poor quality. CC assessments typically use a proper use factor (PUF) or similar coefficients to define this fraction (see e.g. De Leeuw et al., 2019; Petz et al., 2014; Qin et al., 2021). However, we considered it inappropriate to apply a single PUF for all grassland worldwide, because PUFs vary significantly between ecosystems (Fetzel, Havlik, Herrero, & Erb, 2017). Instead, we decided to exploit the minimum (0.20) and maximum (0.65) PUF values reported in the literature (Bornard & Dubost, 1992; De Leeuw et al., 2019; Mayer et al., 2005; Neudert et al., 2013; Vallentine, 2000) to define class boundaries for the RSD.

To examine the livestock‐grazing grasslands separately, on top of the analysis covering all the grasslands, we masked the CC and RSD maps with livestock‐grazing grassland extent from Robinson et al. (2011). In the livestock‐grazing system, 90% of the forage consumed by animals comes from pastures and rangelands. Thus, we can better separate mixed and industrial production systems from grazing and detect overgrazing more reliably than for all grasslands.

We used R version 4.0.4 (RStudio Team, 2021) for the analyses, but processed the land cover classes, NPP, and forest coverage in the Google Earth Engine platform before pulling them into R. As the AGB estimates are negative in areas where the average temperature falls below −13°C (see Equation 1), we excluded areas containing these values from the analyses.

### 2.4 Trend, variability, and uncertainty analyses

Each input data set has its own uncertainties. Thus, combining different global data sets and individual parameters increases the uncertainty that must be considered. Previous global grazing studies have explored the uncertainty by comparing different databases (Fetzel, Havlik, Herrero, Kaplan, et al., 2017; Monteiro et al., 2020) and robustness by testing the sensitivity of the model output for the varying inputs (Petz et al., 2014). In addition, assumptions such as if generally 60% of the NPP is allocated aboveground (Fetzel, Havlik, Herrero, & Erb, 2017; Fetzel, Havlik, Herrero, Kaplan, et al., 2017; Petz et al., 2014; Wolf et al., 2021), or that a feed intake of an animal is a fixed percentage of its body weight (Petz et al., 2014), are decisive for the outcome.

In this study, we accounted for combined uncertainties of the data using the Markov chain Monte Carlo simulation method (see detailed description in Appendix S1). Using the uncertainty parameters reported in input data documentation (Table 2; Appendix S2), we generated distributions for each of the data sets by creating 1000 random values within the appropriate distribution (truncated normal distribution or uniform distribution—see Table 2 and Appendix S2). Finally, we combined simulated data products and derived median maps for AGB and CC (for every year 2001–2015) and RSD (a single map for 2010 based on the median values of CC over 2008–2012) with a resolution of 5 arc‐min (~10 km at the equator). We used the median annual maps for CC to calculate trends over 2001–2015 by using linear regression (timesteps as independent variable, corresponding grid cell values as dependent) as well as interannual variation with the coefficient of variation (CV). In addition, we extracted the annual data to 12 regions and individual countries and examined average trends in CC with Kendall rank correlation. We also derived total AGB and CC sums as well as total grassland areas for individual countries over 2001–2015.

To examine the uncertainty in each grid cell, we calculated the CV at the grid scale for AGB, CC, and RSD based on their standard deviations and means over the 1000 rounds of Markov chain Monte Carlo. Thus, the values we present in this article include all the uncertainties related to the input data, and the CV reflects the dispersion of the simulated distributions.

## 3 RESULTS AND INTERPRETATION

### 3.1 Aboveground biomass and carrying capacity

**FIGURE 2:**
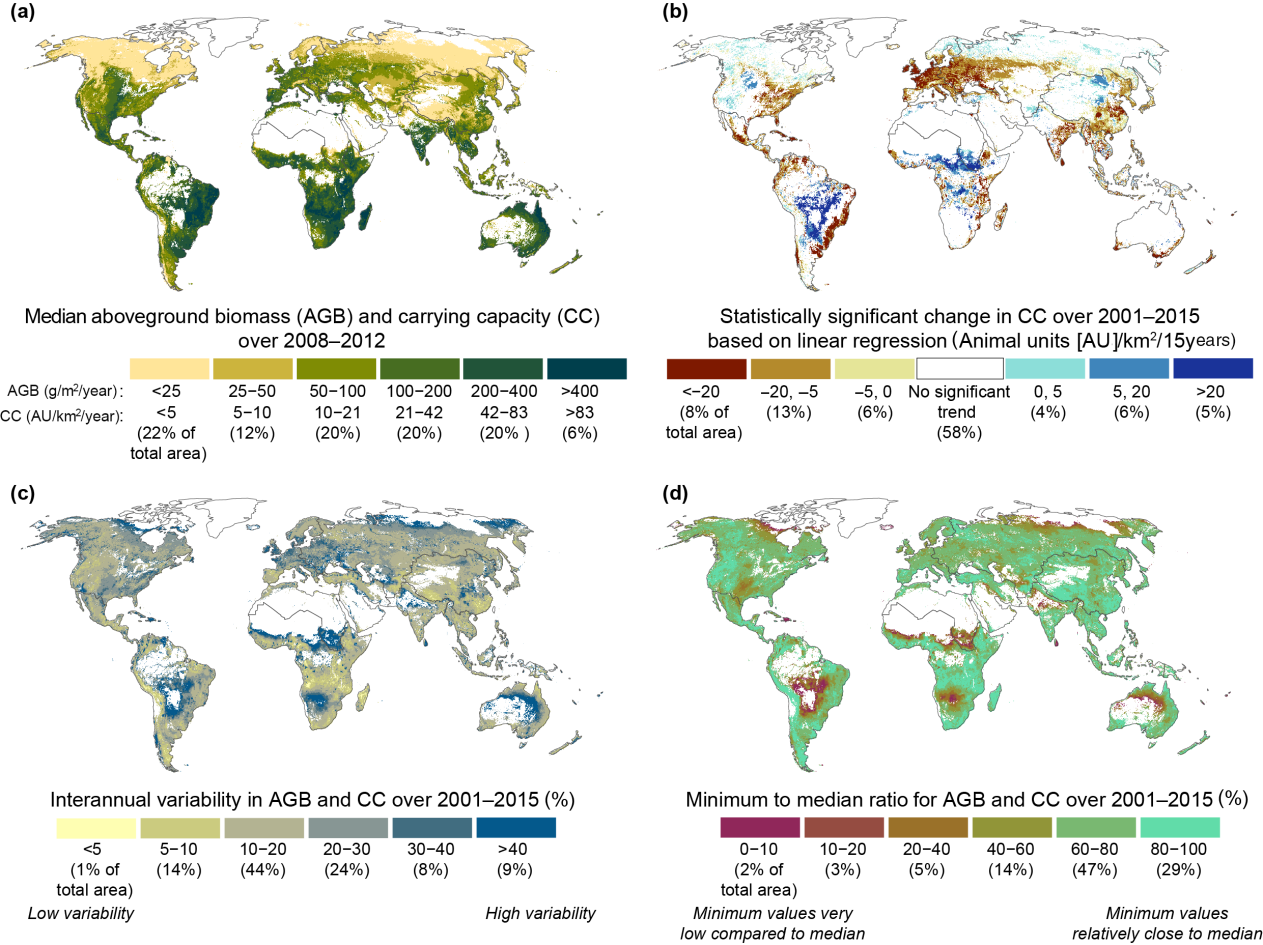
Aboveground biomass (AGB) and carrying capacity (CC) status, trends, and variability. (a) AGB and CC on grasslands showing median AGB and CC over 2008–2012 (first, we calculated median simulated AGB and CC for each year and then selected the median of those five values); (b) the statistically significant trend of CC over 2001–2015 based on linear regression (here we present statistically significant slopes and multiply them by the number of study years (15) to get changes in potential animal units per area [AU km^−2^ 15 year^−1^]); (c) interannual variability of AGB and CC over 2001–2015; and (d) minimum to median ratio of AGB and CC over 2001–2015. All AGB and CC maps presented here are based on yearly median values of Monte Carlo simulated data (*n* = 1000) as presented in methods and in the appendix and masked to areas where AGB is larger than 0.1 g m^−2^ year^−1^. Please note the colors in each map represent the variables over the whole cell area and do not imply the actual grassland area within the cell. For the share of grassland in 5 arc‐minutes cells, see Figure S6a in the Appendix. The fraction of each country's land area covered by grasslands is presented in Figure S6b, and for total grassland area for individual countries, see Figure S6c. See results derived from the ISIMIP2a model ensemble based net primary productivity in Figure S9 in the Appendix

The values of the AGB are greatest in low latitudes where there is also a large geographical variation depending on the humidity/aridity of the climatic zone (Figure 2a). As the CC values were calculated from AGB by dividing it by animal forage requirements per AU, which do not vary geographically, the pattern of the maps for CC as well as trends and variability are similar to those of AGB (Figure 2)—thus, CC results are presented here together with AGB results. In very arid conditions, AGB may fall below 10 g m^− 2^year^−1^, whereas the most productive grasslands in the subtropics and tropics produce biomass over 500 g m^−2^ year^−1^, providing feed (i.e., CC) for more than 100 animal units (AU) km^−2^ year^−1^ (Figure 2a). Notably, large areas of high AGB and CC can be found in the eastern parts of South America and in East Africa (Figure 2a), where the NPP values are also the highest (Figure S2 in the Appendix). Our results in these areas are in line with existing local studies and field observations, such as Fidelis et al. (2013), who collected samples of AGB in South America and found that biomass can yield over 500 g m^−2^ year^−1^ on tropical wet grasslands. Moreover, Cox and Waithaka (1989) collected samples from tropical grasslands in Kenya that even yielded biomass of 1000 g m^−2^ year^−1^.

The median CC over 2008–2012 remains, however, under 21 AU km^−2^ year^−1^ (0.21 AU ha^−1^year^−1^) in the majority (54%) of the areas we consider as grasslands. Due to the low AGB values and dense tree canopy (Figure S3 in the Appendix), the northernmost areas of the globe are incapable of maintaining high stocking densities from the CC perspective. Additionally, steep terrain slopes in mountainous areas especially in Central Asia diminish the grazing possibilities (Figure 2a). Local studies that integrate field survey data and MODIS data sets to derive AGB estimates on Tibetan grasslands (Yang et al., 2009) and on grasslands in Northern China (Zhao et al., 2014) are also in line with our findings, as they report moderately low AGB values (around 70 g m^−2^ year^−1^) in these areas.

Global studies dealing with the NPP partitioning (dividing the NPP into belowground biomass and AGB) are rare, but a recent study (Sun et al., 2021) supports our conclusions that high AGB values appear in Central Africa, Brazil and Northern Australia, whereas AGB decreases with increasing latitudes. For more insights, we calculated country‐specific total AGB estimates (Figure S6d in the Appendix) and compared those with total biomass estimates reported by Sun et al. (2021) and a global study by Wolf et al. (2021). Compared to those studies, our total AGB estimates are equal in size in Argentina, Brazil, and Mexico, but divergent especially in Canada, China, and Russia, where our estimates are lower (Figure S5 in the Appendix). This difference can be explained by our more sophisticated methods in taking the tree cover and terrain slope into account in the AGB calculations (see Methods), as our “unrestricted” biomass estimates are significantly higher than the restricted ones and generally higher than found by Sun et al. (2021) or Wolf et al. (2021; Figure S5 in the Appendix). Our country‐specific total AGB estimates are similar to each other regardless of the used NPP data (MODIS NPP or ISIMIP2a modelled NPP; Figure S5 in the Appendix).

### 3.2 Trends in aboveground biomass and carrying capacity

Unlike the total grassland area (see Figure S6e in the Appendix), AGB, and CC values vary notably between the years, partly due to normal year‐to‐year variation and partly due to longer‐term trends. Indeed, when looking at the statistically significant local trends between 2001 and 2015 (Figure 2b), we see that CC has decreased in Europe, Southeast Asia, the southeast region of Brazil and the East Coast of the United States. Although the trends are insignificant in the majority (58%) of all the grassland areas, the statistically validated trend is more likely negative (27% of the cases) than positive (15% of the cases). Some of the grasslands (8% of total area) have lost more than 20 AU km^−2^ in potential pasturage capacity during the 15 years of study period—for example, with more than a 50% decrease in large parts of Europe (Figure S7 in the Appendix). At the same time, the CC values have increased in some parts of South America and Sudano‐Sahel (Figure 2b) where the IV, measured with the CV, is also notably high (more than 40%, Figure 2c). In addition to this, IV is considerably high (over 40%) in some regions of Australia and in Arctic Russia (Figure 2c). The same areas stand out when comparing the grid cell‐wise minimum values with the median values of 2001–2015 (Figure 2d). This ratio shows that the extreme low AGB and CC values (<20% of the median values) occur most frequently in low latitudes, but also in the arctic regions. Grasslands, where minimum AGB and CC values are relatively close to the long‐term median (minimum values are higher than 80% of the median), cover a bit more than one‐quarter (29%) of the total grassland area.

Although there are no global studies to which we could compare our trend analysis, some local studies examining the trend in CC or in herbage production exist. These have mainly concentrated on China (Cheng et al., 2017; Lyu et al., 2021; Qian et al., 2012; Yang et al., 2018) or Mongolia (John et al., 2018). For example, using other MODIS data than we (MOD09GA) connected to the field measurements, Yang et al. (2018) report an increasing trend or no trend in the Three‐River region in southern parts of Qinghai province, China over 2001–2016, which corresponds to our findings showing an increasing or no trend for AGB in this region. Our findings (Figure 2b) are also consistent with those of John et al. (2018), who used field observations combined with MODIS NBAR product to find an increasing AGB trend on Mongolian Plateau over 2000–2016. In addition, Lyu et al. (2021), who used MOD13Q1 to estimate AGB, find an increasing trend of AGB dominating in southern parts of Inner Mongolia. Qian et al. (2012) examine the trend in herbage production in the main grasslands of China using field observations and modelling and report a positive trend over 1961–2007 in Tibet and Qinghai and southern parts of Inner Mongolia, whereas negative in northern parts of Inner Mongolia, Ningxia, and southern parts of Gansu. Despite the different time span, the patterns of their findings agree rather well with ours, although they show a strong positive trend in Tibet while our results indicate no trend or a weak positive trend.

**FIGURE 3:**
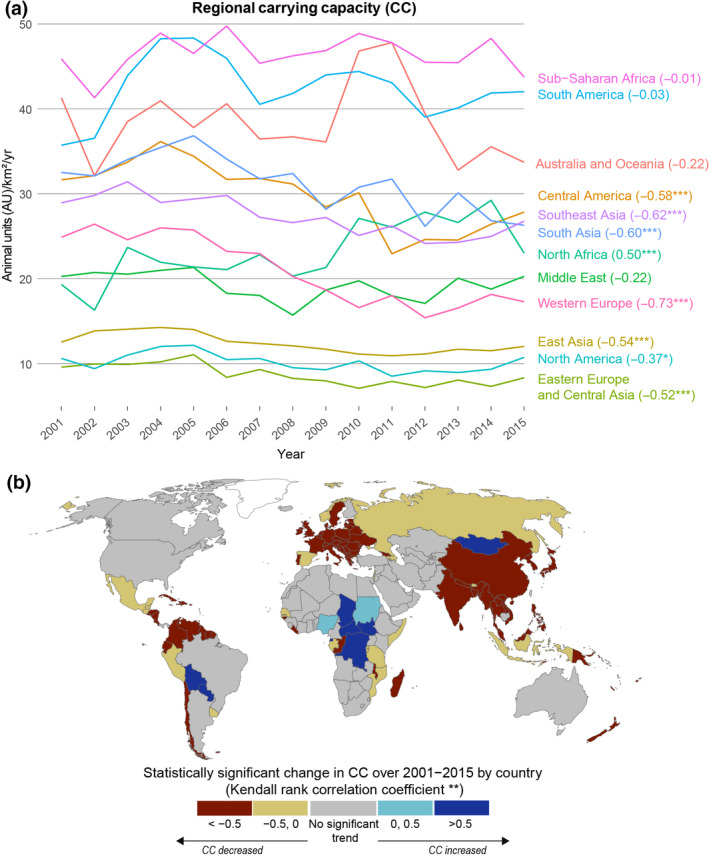
Trends in carrying capacity over 2001–2015 by region (a) and by country (b). Symbols *, ** and *** denote statistical significance levels (related to Kendall rank correlation coefficient) of 10%, 5%, and 1% respectively. Tabulated data for tile (b) is available in the supplementary (Sheet S1)

In addition to the grid cell–wise trend analysis, we also assessed the trend at regional (Figure 3a) and country (Figure 3b) levels. When assessing the average regional CC trends with the Kendall rank correlation test, we found that the trend is insignificant for half of the regions. The trend is negative in regions such as Western Europe and South Asia, but positive in North Africa (Figure 3a). However, the regional and country‐specific trends should be examined with caution as in most regions, such as South America, there are signs of both strong positive and strong negative local trends (Figure 2b). This resulted in insignificant CC trends in many countries such as in Australia, Brazil, Canada, and the United States (Figure 3b). Country‐specific CC trends were strongly negative in most of the European countries, China and India, whereas they were positive in Mongolia and the Democratic Republic of the Congo. The inter‐annual variations in regional CC values are also notable, especially in areas where the CC values are high (Figures 2 and 3a).

### 3.3 Relative stocking densities

**FIGURE 4:**
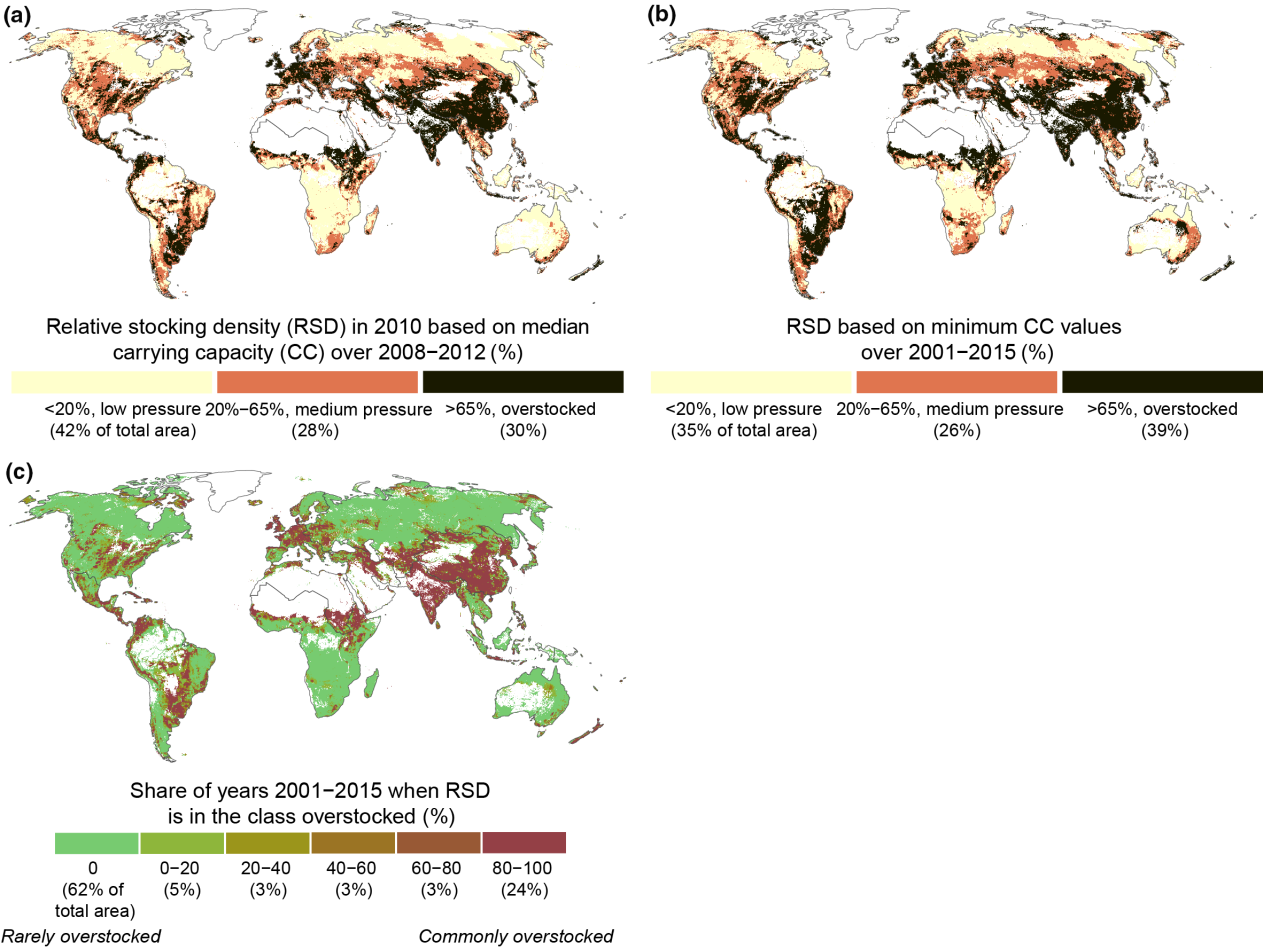
The relative stocking density (RSD), that is, the ratio of carrying capacity (CC) used by the Gridded Livestock of the World (GLW) modelled livestock (industrial and grazing) on the world's grasslands in 2010 based on the median CC of 2008–2012 (for each year, 2008–2012, we calculated RSD by dividing simulated livestock estimates by simulated CC for that year. Then we selected the median of those five annual values) (a). Tile (b) is RSD calculated with minimum CC values and (c) relative shares of years (2001–2015) when the RSD falls in the class overstocked. All RSD maps presented here are based on yearly median values of Monte Carlo simulated data (*n* = 1000) as presented in methods and in the appendix and masked to areas where AGB is larger than 0.1 g m^−2^ year^−1^

We used GLW estimates by Gilbert et al. (2018) to evaluate RSD. Our results show that most parts of the world's grasslands fall either within “medium pressure” (28% of all grasslands) or “overstocked” (30%) categories of RSD (see the Methods section), but the “low pressure” areas also cover considerable parts (42%) of the world's grasslands (Figure 4a). RSD falls within the “overstocked” class (i.e., exceeding grassland CC) in large parts of southern Asia, eastern Asia, northwestern Europe, and the Sahel region (Figure 4a). Overgrazing has indeed been reported to occur in many of these regions, such as in the Three‐River Headwaters region in China (Zhang et al., 2014), in Inner Mongolia (Qin et al., 2021), and in the Patagonian rangelands in Argentina (Gaitán et al., 2018). Most parts of southern Africa, Australia, Canada, and Russia fall, in turn, within the “low pressure” RSD category (Figure 4a). As shown above, in various parts of the world, the available biomass varies considerably between the years (Figure 2c–d). Thus, to understand the pressure on grazing during the years when biomass is low, we assessed how much each location can sustain animals during the year that has the lowest pixel‐specific CC (“minimum CC”) over the study period (2001–2015). In practice, planning the grazing based on minimum CC might also guarantee forage availability during years with low productivity due to climate shock, for example. We found that land area in the “overstocked” category increased from 30% to nearly 40% when minimum CC was used (Figure 4b). In general, areas originally in the “medium pressure” class became overgrazed, whereas in an extreme case, RSD in some parts of Queensland (Australia) jumped directly from “low pressure” category into “overstocked” category (Figure 4a,b). Finally, we calculated RSD using CC for each year and then assessed how many years over the study period an area would fall into the “overstocked” category using the grazing intensity of 2010. We found that most areas that were overstocked using the median CC values over years 2008–2012 (Figure 4a) would be overstocked with each year's CC, and areas in the “low pressure” RSD category remained properly stocked (Figure 4c).

Existing global studies assign many areas a higher grazing potential than the current stocking density (Fetzel, Havlik, Herrero, & Erb, 2017; Monteiro et al., 2020; Petz et al., 2014; Rolinski et al., 2018; Wolf et al., 2021; see Figures S10–S13 in the Appendix). While our estimates (Figure 4) partly align with the abovementioned studies, they are generally more conservative. For example, our results (Figure 4a) agree with Fetzel, Havlik, Herrero, & Erb (2017; Figure S10 in the Appendix) in that the grazing pressure is very high in the Sahel and East Asia, as well as in suggesting that grazing pressure could still be increased in large parts of Sub‐Saharan Africa. However, the results compared with Fetzel, Havlik, Herrero, & Erb (2017) diverge in South America, where our estimates show no potential to increase stocking densities in the grasslands.

Compared with Petz et al. (2014), who found that grazing intensities are low in most of the rangelands, our results are much more conservative. Furthermore, while our results partly align with Monteiro et al. (2020) and Wolf et al. (2021; Figures S11 and S12 in the Appendix) in that Central Africa and Australia fall into the “low pressure” RSD category, we conclude that the actual possibilities to increase grazing in these areas are limited. This pertains to constraints discussed throughout the paper, such as feed shortages during the long dry season, strong year‐to‐year variation in biomass production, tropical livestock diseases and other limiting environmental factors. Regardless of the low stocking density in relation to calculated CC in some areas, many of the grazing lands are near or above their peak livestock (Figure 4).

**FIGURE 5:**
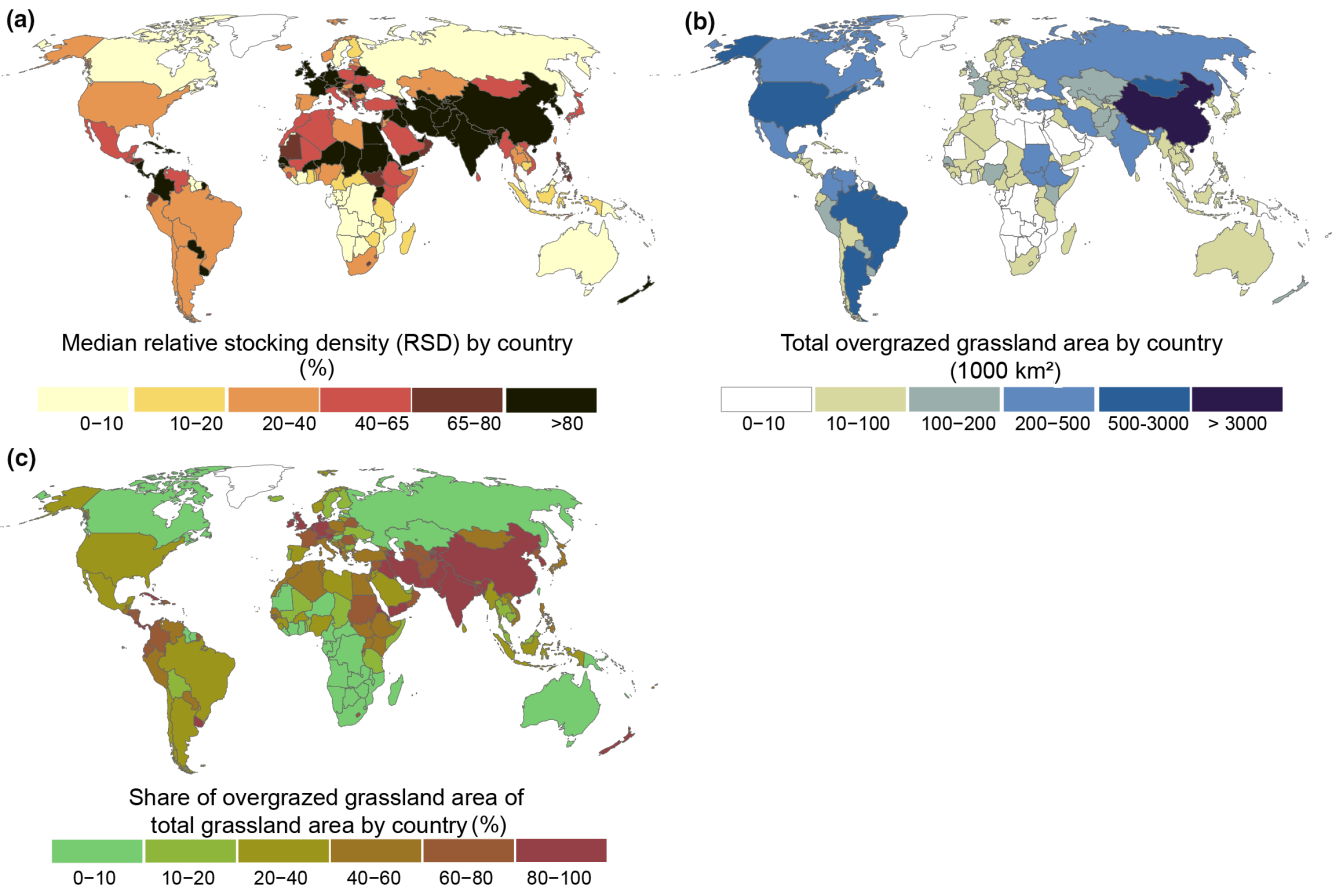
Median relative stocking density (RSD) by country (a), total overgrazed grassland area by country (b) and share of country's grassland area overstocked (RSD > 65%) in tile (c). The RSD in tile (a), that is, the ratio of carrying capacity (CC) used by the Gridded Livestock of the World (GLW) modelled livestock (industrial and grazing) on the world's grasslands in 2010 based on the median CC of 2008–2012 (for each year, 2008–2012, we calculated RSD by dividing simulated livestock estimates by simulated CC for that year. Then we selected the median of those five annual values) and extracted median values by countries. Tiles (b, c) are also based on the median CC of 2008–2012. Tabulated data for this figure is available in the supplementary (Sheet S2)

For further illustration of overgrazing severity by country, we calculated country‐specific median RSD (Figure 5a), total overgrazed area (Figure 5b) and the share of overgrazed grassland (Figure 5c). For example, grasslands of China, India, Pakistan and Iran suffer from heavy overgrazing (RSD > 80%; see Figure 5a) and overgrazed grassland areas cover over 80% of the total land area of these countries (Figure 5c). Overgrazing, measured with RSD, is most visible in China, where the sum of overgrazed grassland areas exceeds 3 million km^2^ (Figure 5b). In countries where our total biomass estimates are much lower than expressed in other studies (e.g., China, see Figure S5 in the Appendix), there might be somewhat less pressure on grasslands than what we report here (Figure 5a). However, these studies (see Figure S5 in the Appendix) are not directly comparable due to different methods as well as grassland extent, as discussed in Section 4.2.

### 3.4 Carrying capacities and relative stocking densities in the production system “livestock‐grazing”

**FIGURE 6:**
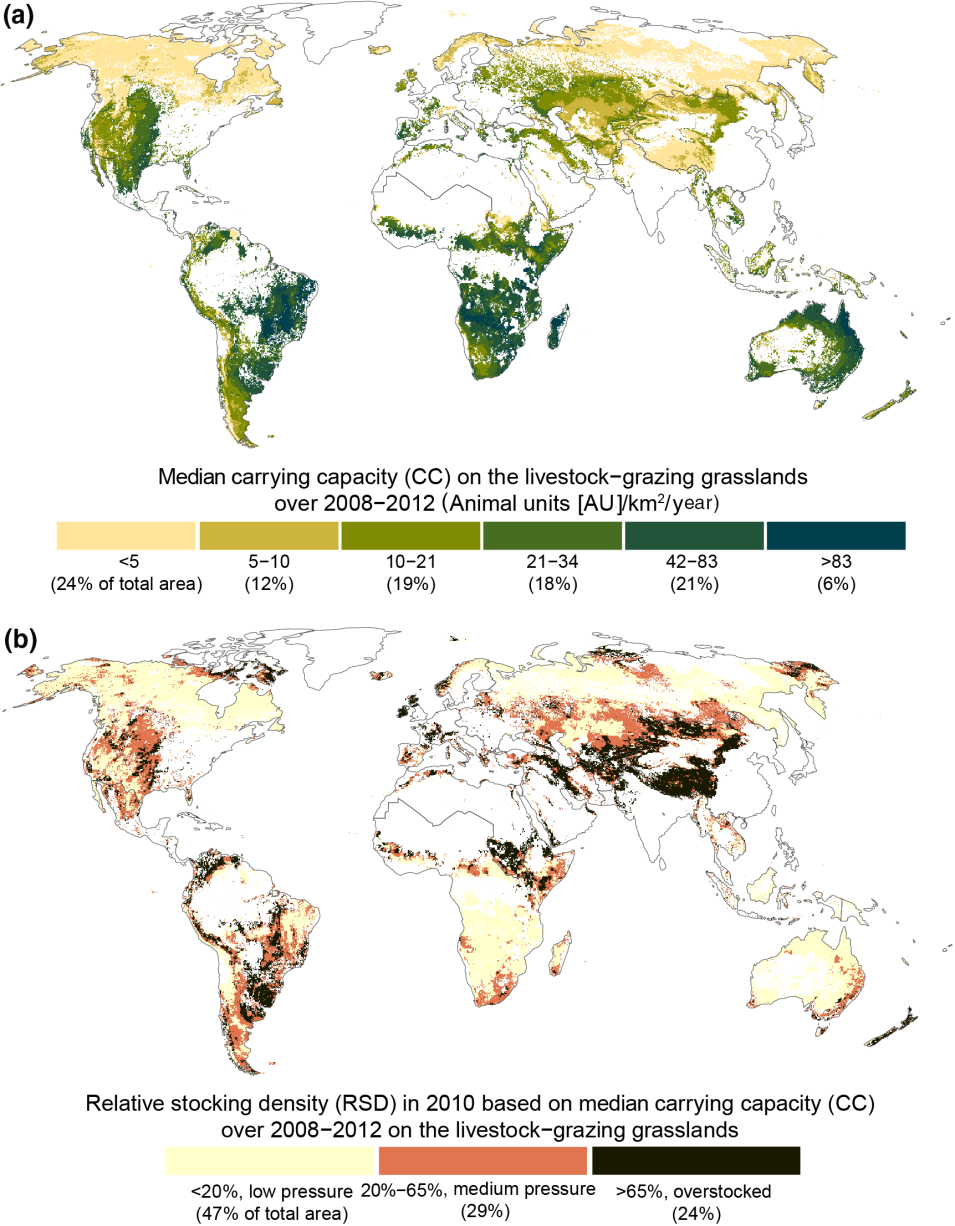
The carrying capacities (CCs) (a) and relative stocking densities (RSDs) (b) on grasslands with a production system of “livestock grazing.” First, we calculated the median CC for each year and then selected the median of those five values (a). For (b) we calculated RSD by dividing simulated livestock estimates by simulated CC for that year. Then, we selected the median of those five annual values. Finally, we masked both maps (a, b) to “livestock grazing” grasslands. All maps presented here are based on Monte Carlo simulated data (*n* = 1000) as presented in methods and in the appendix and masked to areas where aboveground biomass is larger than 0.1 g m^−2^ year^−1^

Our calculations do not consider consumed supplementary feed or account for all the variations in production systems ranging from extensive to intensive farming. Therefore, we estimated CCs and RSDs only on grasslands where the production system is categorized as “livestock‐grazing” (Herrero et al., 2013; Robinson et al., 2011; see the Methods section). According to our simulated AU estimates (see Appendix S2.8), approximately 629 million AUs inhabit these livestock‐grazing grasslands, which covers around 42% of AUs (i.e., animal units) grazing all the grasslands. The potential CCs on these livestock‐grazing grasslands (Figure 6a) are relatively high, and the average RSD on these areas is 56% – much lower than the calculated 97% over all grasslands (see Appendix S2.8). This average global RSD (97%) is inflated because the AUs on grasslands outside the livestock‐grazing areas rely largely on supplementary feed. However, overstocking seems to be less common on livestock‐grazing systems than on all grasslands together, albeit more important, as most of the forage comes from pastures and rangelands.

Our livestock‐grazing RSD map (Figure 6b) shows that arid regions such as southern Africa and Australia mostly fall into the “low pressure” category. However, our results diverge from those of Monteiro et al. (2020), who observe that grazed‐only systems perform below their potential especially in arid areas. Instead, we argue that increasing stocking densities in these areas is highly questionable, as discussed in Section 4.1.

### 3.5 Uncertainty estimates

**FIGURE 7:**
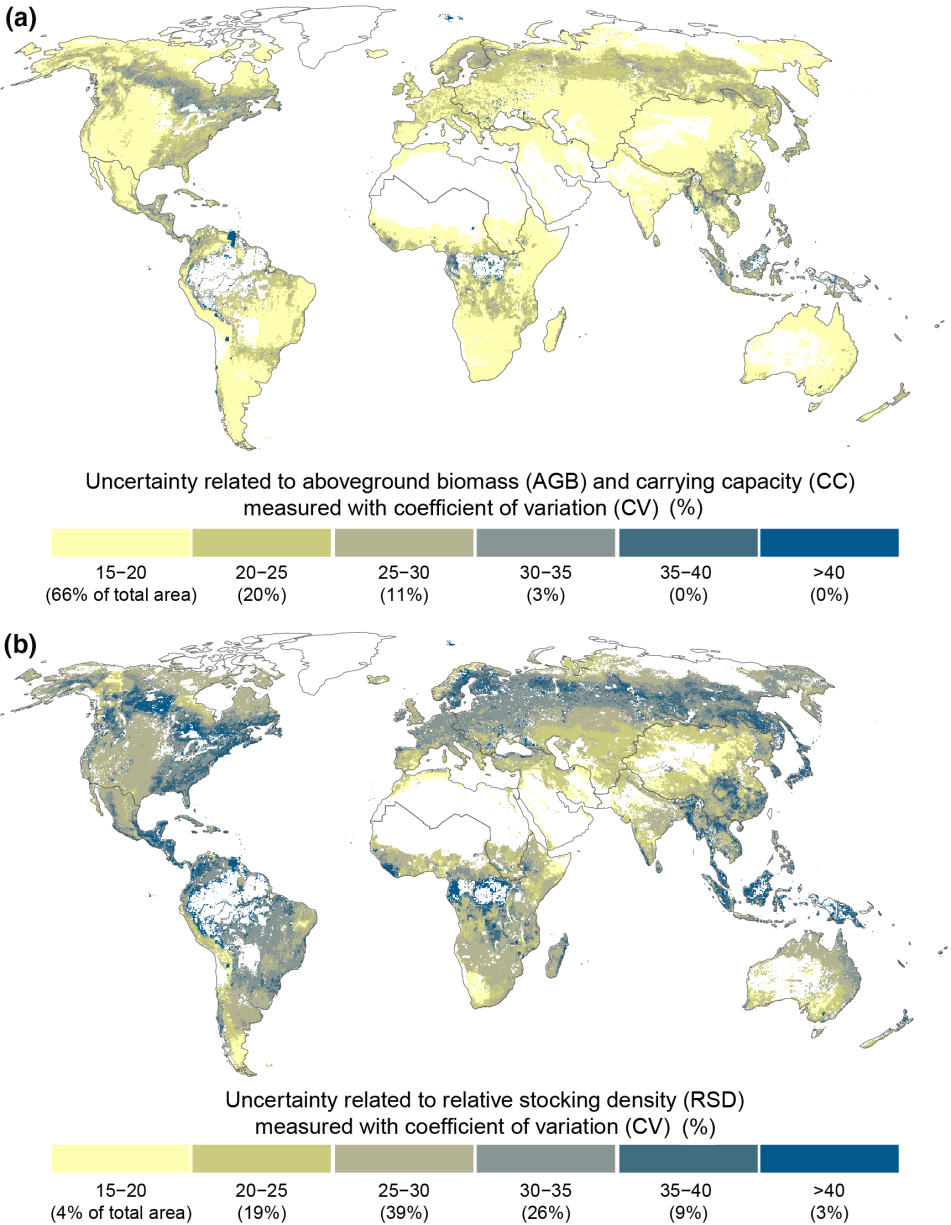
Uncertainty of aboveground biomass (AGB) and carrying capacity (CC) (a) and relative stocking density (b) originating from uncertainties in input data, measured with the coefficient of variation (CV). First, we calculated the CV for each year and then selected the median of those five CV values. The CVs for individual years were calculated from Monte Carlo runs (*n* = 1000). See uncertainty results of the AGB derived from ISIMIP2a model ensemble based NPP in Figure S9 in Appendix

We assessed the uncertainty of our AGB, CC, and RSD estimates by calculating the CV over the 1000 Monte Carlo runs (see Methods). The uncertainties related to AGB and CC were moderate (between 15% and 20%) in two‐thirds of the grassland areas, especially near the Tibetan plateau and on the Great Plains (Figure 7a). In general, dense tree canopy coverage seems to increase the uncertainty (see Figure S8c in the Appendix), as the uncertainty of AGB and CC is relatively high (more than 25%) particularly in boreal forests (Figure 7a). The magnitude of uncertainty is equal to Sun et al. (2021), who found CV values related to AGB ranging from 3% to 25%. Uncertainties related to RSD estimates (Figure 7b) are much larger than uncertainties related to AGB or CC estimates (Figure 7a). The RSD values fluctuate over 25% in most (around 77%) of the grassland areas, and the uncertainties are at the same level with the AGB and CC (below 20%) only in marginal areas (4% of all the grasslands). This is due to the high uncertainty related to different animal unit (AU) conversion factors linked to GLW modelled livestock estimates (see Appendix S2.8 and Figure S8d in the Appendix).

For a comparison, we also calculated AGB using modelled NPP data (an ensemble of four models) derived from the Inter‐Sectoral Impact Model Intercomparison Project Phase 2a (ISIMIP2a—see Reyer et al., 2019). Compared with AGB derived from MODIS NPP product (based on remote sensing), the AGB based on modelled NPP data resulted in a rather similar outcome (Figure S9d in the Appendix). The largest differences can be seen in some rather dry areas (e.g., Sahel, Tibetan plateau) where modelling‐based AGB resulted in larger values than MODIS‐based, whereas in some wet areas (e.g., eastern Brazil, southern Congo Basin) the opposite was the case. At a country level, the results derived from these two products agree rather well (Figure S5 in Appendix). In addition, uncertainty in modelled AGB results (Figure S9c in the Appendix) was greater than the uncertainty related to MODIS‐derived ones (Figure 7). The detailed procedure for estimating the AGB using multiple models can be found in Appendix S2.10.

## 4 DISCUSSION AND CONCLUSIONS

This study estimated, for the first time, the global trend and IV in AGB and CC over the recent past. We showed, for example, that CC decreased considerably in large parts of Europe and Asia, while the trend was positive in parts of South America and sub‐Saharan Africa (Figure 2b). In addition, we found large interannual variation in various locations (Figure 2c), directly impacting potential overstocking (Figure 4), measured here with RSD. Our uncertainty analysis, done with the Markov chain Monte Carlo method, revealed areas with high uncertainty in the CC and RSD estimates (Figure 7), as well as some indications of the most sensitive variables impacting the uncertainty (see Figure S8 in the Appendix).

Our approach provides a method—based on open‐access global data sets—to conduct continuously updated estimates of global gridded AGB, CC, and RSD. Timely estimates are crucial, as the herbaceous biomass of rangelands is estimated to decrease in many regions toward the 2050s (Godde et al., 2020). Decreasing rangeland biomass, combined with the increasing inter‐annual variability of climate and forage, will create notable challenges to livestock management across the world's grasslands, with implications for food production, human welfare, and ecosystem resilience. The optimal density of livestock will significantly change depending on the region, which may call for the re‐optimization of livestock distribution.

### 4.1 Factors impacting on carrying capacity and relative stocking density values

We acknowledge that available grass biomass is only one factor impacting the CC of animal production. Other factors include, for example, feed shortages during the long dry season and strong year‐to‐year variation in biomass production in the arid and semiarid zones (Vetter, 2005). Furthermore, C4 grasses—dominating the tropical grasslands—have lower nutritional value and are poorly digested by ruminants compared to C3 grasses that are dominating the temperate zone grasslands (Barbehenn et al., 2004). In addition, animal diseases (e.g., tsetse in southern Africa), poisonous plants, and often scarce water resources limit the grazing possibilities in tropical grasslands (De Leeuw et al., 2001; Holechek et al., 2010). In arctic and temperate continental grasslands, feed shortage or difficulty accessing forage during long winter periods control livestock populations (Hui & Jackson, 2006; Suttie et al., 2005). Due to these constraints, the potential to increase livestock grazing is lower than suggested by our RSD map (Figure 4a) in many places. Given the above constraints, exceeding the upper boundaries of CC can be extremely harmful, and increasing the stocking densities even in the medium‐pressure regions might lead to land degradation. At the same time, other unaccounted factors may support higher grazing rates in certain regions than those introduced in our study. For example, in regions such as the Sahel, livestock migrates seasonally between rainy season and dry season pastures (Dixon et al., 2019; Yi et al., 2008), which has an impact on animal densities, and thus, on grazing pressures at a local level. We recognize that further rewilding efforts require setting aside space and biomass for wild grazers, as they are competing with the same resources with grazing livestock.

Although the existing livestock leaves some of the biomass untouched (“low pressure” category) in the northernmost areas of the globe, those areas have limited potential for increased grazing due to low biomass availability (Figure 2a). Nonetheless, higher animal densities can be sustained in regions where livestock is not only fed with locally produced grass but also externally acquired forages, crop grains, crop residue leftovers, or other feed supplements. Another option is to apply higher inputs to grassland management in the form of irrigation or fertilization where it is feasible. It should be noted that currently, intensive livestock production uses other feed resources produced elsewhere (Naylor et al., 2005), which enables concentrating production on areas where the NPP is low and grazing animals may exist in areas unsuitable from the CC perspective. Large dairy industries in Saudi Arabia, Syria, and Jordan that are not visible in our maps, as those are not located in grassland areas, provide examples of this (Alqaisi et al., 2010). According to Wolf et al. (2021), the amount of total livestock intake requirement supplied by grazing remained around 63% over 2001–2010. Although regional differences in this intake are notable (Sandström et al., 2021; Wolf et al., 2021), supplementary feed generally covers around one‐third of the total livestock intake. Thus, areas where the forage demand of the livestock exceeds the AGB (Figure 4) are most likely dependent on the supplementary feed.

### 4.2 Differences between global grazing studies

In general, global results on grazing pressures differ between studies. According to Irisarri et al. (2017), estimated grazing intensities reported in the literature are generally higher than those modelled by Fetzel, Havlik, Herrero, & Erb (2017), and thus the potential to increase grazing is more limited than suggested by Fetzel, Havlik, Herrero, & Erb (2017). Our results support this, as we found that the stocking densities are already high or above the over‐stocking limit in various places (Figure 4, Figure 5a). Divergent results between global studies can be explained, at least partly, by different methods; global simulation models, such as JULES and ORCHIDEE, yield different NPP estimates compared to field data or the MODIS NPP product in many places (Chang et al., 2015; Slevin et al., 2017). Nonetheless, the AGB results we derived using modelled NPP data (Figure S9a in the Appendix) were rather similar compared with the MODIS NPP derived ones (Figure 2a), with certain differences (see Section 3.5).

The method we developed, applied to both MODIS‐based and modelled NPPs, might partly explain the differences between our results and existing literature. While we used temperature as a predictor when allocating a fraction of total NPP to AGB, other global grazing studies, either modelled (Fetzel, Havlik, Herrero, & Erb, 2017) or MODIS‐based (Petz et al., 2014; Wolf et al., 2021), use a single constant value of 0.60 (i.e., AGB =0.60 * NPP) to derive the aboveground fraction from NPP. Moreover, restrictions of terrain slope and tree cover applied in this study (see Methods)—which were not used in other global studies—significantly impacted our AGB estimates (see Figure S5 in the Appendix), and thus the resulting RSD. The definition of grazing land also notably differs between the studies because different land cover maps produce varying estimates of land cover type (Fritz et al., 2011). In addition, uncertainties related to modelled livestock distributions and different animal unit conversion factors have an impact on estimated grazing intensities, as shown earlier (see Section 3.5.). Given the above, comparison of different global studies of grazing intensities is always difficult when there is no common protocol, as methods, input data, and assumptions differ greatly. Still, all these modelling and MODIS‐based estimates complement each other and increase the understanding of the topic. Our work with improved methodology, detailed uncertainty analysis, as well as IV and trend analyses provide new valuable insights at both local and global levels.

### 4.3 Limitations and future directions

Here, we analyzed the yearly NPP values over a period of 15 years to obtain robust estimates of the available feed. Our results thus represent average conditions within a year, but monthly estimations of CC would also be needed when defining proper stocking rates (without supplementary feed) in a highly variable climate. While our method can be used to produce these seasonal estimates, they were beyond the scope of this study and thus left for future analyses.

The method we used, based on satellite products, enabled the global analysis over various years, but it naturally has limitations in detecting some specific conditions on the ground. For example, poor feed quality or dead biomass still cannot be observed by current satellite products. Therefore, we suggest that our global grazing‐related maps should be verified based on local observations and knowledge—on top of the validation we conducted in the Results section comparing our findings to existing studies. Furthermore, the satellite spectrometers cannot measure the sub‐canopy vegetation that is available for grazers in forest areas; thus, we needed to develop a function (Equation 2, see Appendix S2.4) to translate the tree canopy cover into the fraction of NPP that is allocated to the understory. The estimated AGB may, therefore, differ from reality—especially in woody areas, whose understory forage yields depend heavily on different tree species and forest type. Future studies should, therefore, improve the transfer function (Equation 2) to observe this factor linked to forest canopies – for example, by creating a separate function for different forest types. Similarly, the satellite products are not able to detect the differences between the plant species, and thus, we were not able to determine the specific quality of the feed in each location. Future studies could potentially enhance this by detecting specific species in each location to better estimate the forage requirements. In addition, as in previous studies, we did not consider the response between grazing and the NPP values, meaning that we did not account for livestock trampling that might have impacted the potential NPP values in some regions. Future research could improve the methodology by trying to estimate this feedback in the analysis.

We used the MODIS land cover map (Sulla‐Menashe & Friedl, 2018) to determine the grasslands. Although this product is widely used (e.g., Xie et al., 2019), more precise land cover and NPP maps would improve the accuracy of the results (Erb et al., 2016). However, identifying the location of grazing lands based on land classification systems will likely pose challenges even in the future, due to the significant differences between land cover maps, as discussed by Fetzel, Havlik, Herrero, Kaplan, et al. (2017) and Fritz et al. (2011). Ideally, the land cover classification maps that divide the land area into pastures, grasslands, and other types should not restrict the CC assessment. Instead, other restrictions—such as soil erosion, wood cover, or quality of the feed—should influence the determination of suitable grazing areas for livestock. Moreover, improved methods in biomass partitioning (dividing the NPP into belowground biomass and AGB) would increase the accuracy of the grazing studies (see Sun et al., 2021).

In addition to the environmental sustainability perspective given here, economic and social sustainability perspectives should also be considered when optimizing livestock production (Steinfeld et al., 2013). This could be done by detecting the opportunity cost of grazing in different areas, and then indicating where the economically sustainable intensification of grassland is feasible in the first place. This examination could, for example, result in dividing grasslands into arable and non‐arable grasslands and determining where grazing livestock does not compete with crop production.

### 4.4 Concluding remarks

We conducted an improved satellite observation‐based global CC estimate of the world's grasslands over 2001–2015. With the continuous time series at grid scale over these years, we were able to provide much‐needed insights on global trends in and interannual variation on AGB and CC. Our findings support the planning of more sustainable land management policies on grazing areas. They can therefore help to identify both undergrazed areas for targeted sustainable intensification efforts, but even more importantly, assist with conservation efforts to reduce land degradation associated with overgrazing. Although our results imply that nearly 60% of the world's grasslands fall within the “medium pressure” or “overstocked” categories of RSD, large areas still belong to the “low pressure” category. However, we argue that grazing densities may not be sustainably increased in all these “low pressure” regions. Areas with such unused capacity with limited production value could be better used for ecosystem services, carbon sinks, or rewilding (Carver, 2019; Navarro & Pereira, 2012). Alarmingly, we also found that various parts of the globe have experienced strong negative trends in CC, as well as high interannual variation further impacting the assessment of the sustainability of current grazing practices. Finally, the method we developed allows updating these estimates regularly on an annual or even on monthly basis; thus, the method provides a tool that can be used to continuously assess the changes in AGB and CC globally.

## CONFLICT OF INTEREST

The authors declare no conflicts of interest.

## Supporting information

Supplementary Material

## Data Availability

All input data (excluding “livestock‐grazing” grasslands mask) and relevant output data are available at: https://doi.org/10.5281/zenodo.6366896. All source codes are available in GitHub: https://github.com/jpiippon/cc_rsd_repo.
