# Supplementary material for: Global trends in grassland carrying capacity and relative stocking density of livestock
Source: Glob Chang Biol. 2022 Apr 6;28(12):3902–19. doi: 10.1111/gcb.16174 (PMC9321565; doi:10.1111/gcb.16174)
Supplement: Supplementary file 1 — Supplementary Material [file GCB-28-3902-s001.pdf]

Appendix

Contents

S1. Uncertainty analysis 1
S2. Ranges for different variables and parameters 2
S2.1 Forage requirement – truncated normal distribution [0.018, 0.04] with mean = 0.02 2
S2.2 Carbon conversion factor – truncated normal distribution [0.47, 0.50] 2
S2.3 MODIS NPP – rnorm(1000, mean = 1 , sd = 0.07) 2
S2.4 TreeCoverMultiplier – truncated normal distribution [bottom 2.5%, median and top 97.5% curves] 2
S2.5 fANPP – rnorm(1000, mean = 1 , sd = 0.198 * 0.71) 4
S2.6 Temperature 4
S2.7 SlopesMultiplier 5
S2.8 AU conversion factors – truncated normal distribution 5
S2.9 GLW – DA method 6
S2.10 Aboveground biomass (AGB) based on ISIMIP2a NPP data 6
S3 Other supplementary figures 7
S4 References 14

S1. Uncertainty analysis

We assessed the combined uncertainties using the Markov Chain Monte Carlo method. We searched the literature to choose the most suitable ranges for each of the input parameters and variables used in the analysis. These ranges are used in Monte Carlo simulation when performing uncertainty analysis. We assumed truncated normal distributions for the forage requirements of the animals, carbon conversion factor, animal conversion factors and the TreeCoverMultiplier as presented in the main text (Table 2). We assumed uniform distributions for NPP and fANPP. The distributions were based on 1000 generated observations within the range. Finally, we calculated median values and coefficients of variation for each cell from the Monte Carlo simulated data (n = 1000). The detailed process can be found in the R scripts provided.

S2. Ranges for different variables and parameters

S2.1 Forage requirement – truncated normal distribution [0.018, 0.04] with mean = 0.02

According to Singhal et al. (2005), forage requirements of animals vary between 1.8–4.0% of their body weight, but daily dry matter intake of 4% applies only to some specific goats. Holechek et al. (2010) suggest that 2.0% is suitable for most ruminants, whereas De Leeuw and Tothil (1990) estimate that the range is 2.5–3.0% and Paul et al. (2003) suggest a range of 2.46 – 3.47%. Therefore, we used the maximum range [1.8, 4.0] found in literature when generating truncated normal distribution for animal forage requirements but used forage intake of 2.0% as a mean. The median value of 1000 simulations was used for every cell. In other words, animal forage requirements do not vary geographically. In addition, we calculated animal forage requirements for an average animal unit (AU) that weighs 455kg and for an average year, meaning that we multiplied the simulated forage requirement distribution by 455 * 365.

S2.2 Carbon conversion factor – truncated normal distribution [0.47, 0.50]

Various studies use a carbon conversion factor of 0.50 (e.g. Fetzel et al., 2017; Petz et al., 2014; Saatchi et al., 2011) but other studies use a carbon conversion factor of 0.47(e.g. De Leeuw et al., 2019; Eggleston et al., 2006). Based on this, we created a truncated normal distribution ranging from 0.47 to 0.50.

S2.3 MODIS NPP – rnorm(1000, mean = 1 , sd = 0.07)

We used a similar procedure as Keuper et al. (2020), who assumed that the values of Gross Primary Productivity might vary 7% (Beer et al., 2010). We multiplied 1000 simulated (and normally distributed with 7% variation) values by the cell value to build in MODIS NPP product uncertainty, which we use, amongst other variables, when creating median maps for the carrying capacity (CC). We assumed that the simulated MODIS NPP distribution is the same in each cell, meaning that it does not vary geographically.

S2.4 TreeCoverMultiplier – truncated normal distribution [bottom 2.5%, median and top 97.5% curves]

We digitised the data points found in the studies of Le Brocque et al. (2008, p.11) and Lloyd et al. (2008, p.8) with help of WebPlotDigitizer software. Then we fitted a nonlinear curve to the points (Figure S1) and performed 1000 bootstraps to derive a confidence interval (CI) of 95% for 1000 generated curves. The geographical extent of these studies includes Australia, Brazil and Ghana. Unfortunately, scatter plots including data from high latitudes are not available. Still, we assume that the curve also reflects the tree canopy cover response to consumable biomass in temperate and polar zones.

A single tree can substantially reduce the amount of consumable biomass in the original pixel as the pixel size is very small (30m*30m). Therefore, a steep slope at the beginning of the curve (see Figure S1) makes sense: even a small tree canopy cover % can result in a significant reduction in aboveground biomass that is available for grazers. Or, in other words, a single tree can notably contribute to the

total NPP of the cell; thus, animals can eat only a certain fraction of the total NPP allocated to it. In addition, in our study area (defined in the Methods section) the tree canopy cover maximum is 60% (Sulla-Menashe and Friedl, 2018). Therefore, the rest of the curve, where the Tree Canopy cover exceeds 60%, is not relevant for this study.

Previous grazing studies have usually excluded woody areas from the analysis. For example, Fetzel et al. (2017) excluded areas with tree cover above 30% and reduced consumable NPP to 15% in areas where trees were observed. Similarly, De Leeuw et al. (2019) excluded areas with tree cover above 50%. Our approach is more flexible, and we are aware of uncertainties related to the equation. For example, in savanna ecosystems trees may facilitate the production of grasses in the understory, because of the hydrologic lift of water (Priyadarshini et al., 2016). This means that the consumable biomass might also increase with increasing tree canopy cover.

As explained in the article, we reclassified the original tree canopy cover pixels (Sexton et al., 2013) three times according to the function (Eq. 2)(Figure S1) before aggregating the data into MODIS resolution. The original data was reclassified based on the bottom 2.5%, median and top 97.5% TreeCoverMultiplier curves (see black curves of Figure S1). Thus, we created three different TreeCoverMultiplier rasters, as these rasters yielded 95% CI for each pixel and helped us to define a range (and truncated normal distribution) for each individual pixel.

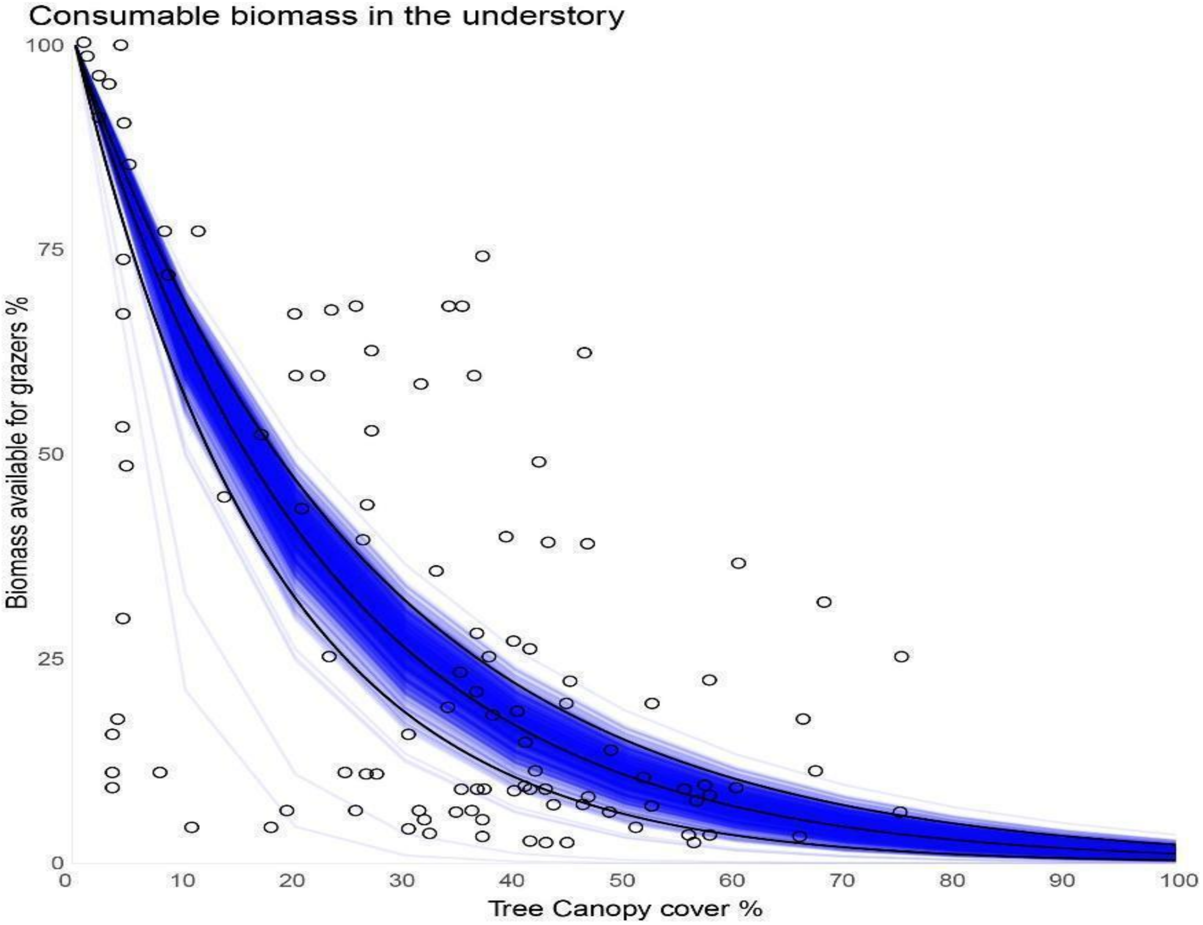

Figure S1. TreeCoverMultiplier function: effect of the tree canopy cover on the biomass available for grazers with 95% confidence interval based on 1000 bootstraps.

To illustrate this, let's assume an area where the tree canopy cover is 0%, 20%, 20% and 30% in adjacent cells (figure/table below). These values are reclassified based on three functions: $TreeCoverMultiplier_{2.5\%}$, $TreeCoverMultiplier_{median}$ and $TreeCoverMultiplier_{97.5\%}$ and then aggregated to MODIS projection. Thus, these aggregated rasters give ranges we can use when creating truncated normal distribution for each pixel.

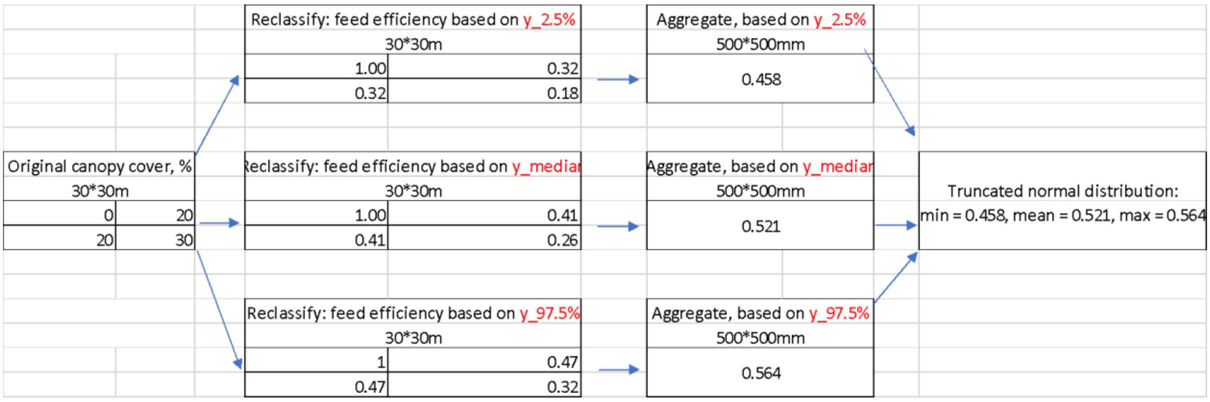

Example of the process of creating the three TreeCoverMultiplier rasters.

S2.5 fANPP – rnorm(1000, mean = 1 , sd = 0.198 * 0.71)

According to Hui and Jackson (2006) fraction of belowground biomass (BNBB) of NPP is

$$f_{BNPP} = 0.829 - 0.0129\ MAT,$$

where MAT is the Mean Annual Temperature in ℃. However, as NPP is the sum of the aboveground and belowground biomass (ANPP + BNPP = 1), the equation for the aboveground fraction of the biomass is

$$(1)\ f_{ANPP} = 0.171 + 0.0129\ MAT$$

According to Hui and Jackson (2006; p.3), interannual variability (noted as CV) for all their study sites is 19.80% and the mean is 0.71. As CV = SD/mean, we can conclude that SD = 0.198 * 0.71 = 0.14058. We use the same simulated normal distribution for each cell to define how much the values of the Eq. (1) fluctuates.

S2.6 Temperature

We assume that the CV and mean related to Eq. (1) already capture fluctuations related to temperature. Hence, SD for temperature has not been assessed separately. However, we used annual average temperatures for each year from 2001 to 2015 when calculating the fraction of aboveground biomass.

S2.7 SlopesMultiplier

We use the classes proposed by George and Lyle (2009) to reduce aboveground biomass (AGB) on steep slopes. The same classes have been used in other studies as well (De Leeuw et al., 2019; Holechek et al., 2010) and as we could not find other reliable alternatives for reduction factors we decided not to account for uncertainties related to SlopesMultiplier.

S2.8 AU conversion factors – truncated normal distribution

Based on the literature below (Table S2), we selected the extreme ranges for conversion factors for different animal units (AU). Truncated normal distribution was assumed for all the AU conversion factors. In addition to min and max values, mean value 1.0 was used for cattle as it is the most commonly used value for converting cows to animal units in the literature. For other species truncated normal distribution was created based on min and max values only. Calculations were based on the modelled distribution of different animal species we extracted from the Gridded Livestock of the World (GLW 3) database (Gilbert et al., 2018). The animal unit conversion factors used in the uncertainty assessment are as follows:

Cattle [0.50 , 1.25] with mean = 1
Buffalo [0.60 , 0.70]
Sheep [0.10 , 0.15]
Goat [0.10 , 0.15]
Horse [0.40 , 1.80]

Table S1. Total number of animal units (AU) based on our simulations and AU conversion factors and total potential carrying capacity (CC) allocated to all the grasslands and livestock-grazing grasslands.

	Million animal units (AU)
Total number of animal units (all the grasslands)	1492*
Total number of animal units (livestock-grazing grasslands)	629
Total potential carrying capacity (all the grasslands)	1537
Total potential carrying capacity (livestock-grazing grasslands)	1117

*Note that animals outside the livestock-grazing grasslands also consume supplementary feed

Table S2. Ranges for animal unit (AU) conversion factors found in the literature

	FAO, 2011: Annex 1	Holechek et al., 2010	Glossary:Livestock unit (LSU) - Statistics Explained," n.d.	Vallentine, 1965
	**Range**	**Range**	**Range**	**Range**
**Cattle**	0.50; 0.55; 0.60; 0.65; 0.70; 0.90; 1.00	1.00	1.0; 0.80	1.00; 1.25
**Buffalo**	0.60; 0.70			
**Sheep**	0.10	0.15	0.10	
**Goat**	0.10	0.10	0.10	
**Horse**	0.40; 0.50; 0.56; 0.60; 0.65; 0.80	1.80	0.80	

S2.9 GLW – DA method

We decided to use the dasymetric method (Da) of Gridded Livestock of the World (GLW) data in the main analysis as the future versions of the GLW will use this method as well. However, we checked that areal weighted (Aw) GLW products yield similar Relative Stocking Density (RSD) estimates (see Figure S5).

S2.10 Aboveground biomass (AGB) based on ISIMIP2a NPP data

As described in the main text, we used observed MODIS NPP data to derive AGB for global grasslands. However, for comparison we also estimated AGB using NPP data from the Inter-Sectoral Impact Model Intercomparison Project Phase 2a, (ISIMIP2a, Reyer et al., 2019) which provides modelled NPP data over 2000-2010 at 30 arc-min (0.5°) resolution. We used four terrestrial biosphere models (CARAIB; DLEM; LPJmL and ORCHIDEE) that were driven by three common historical forcings; GSWP3 (Global Soil Wetness Project 3), PGMFD v2.1 (Princeton), and WATCH-WFDEI (EU WATCH project). Thus, we had 12 models altogether. The individual datasets used are listed in Table S3.

Table S3. NPP datasets from the Inter-Sectoral Impact Model Intercomparison Project Phase 2a, (ISIMIP2a) used in the analysis.

Model	Forcing	Dataset file
CARAIB	GSWP3	caraib_gswp3_nobc_hist_varsoc_co2_npp_global_monthly_1971_2010.nc4
CARAIB	PGMFD v2.1	caraib_princeton_nobc_hist_varsoc_co2_npp_global_monthly_1971_2012.nc4
CARAIB	WATCH-WFDEI	caraib_watch-wfdei_nobc_hist_varsoc_co2_npp_global_monthly_1971_2010.nc4
DLEM	GSWP3	dlem_gswp3_nobc_hist_varsoc_co2_npp_global_monthly_1971_2010.nc4
DLEM	PGMFD v2.1	dlem_princeton_nobc_hist_varsoc_co2_npp_global_monthly_1971_2012.nc4
DLEM	WATCH-WFDEI	dlem_watch-wfdei_nobc_hist_varsoc_co2_npp_global_monthly_1971_2010.nc4
LPJmL	GSWP3	lpjml_gswp3_nobc_hist_varsoc_co2_npp_global_monthly_1971_2010.nc4
LPJmL	PGMFD v2.1	lpjml_princeton_nobc_hist_varsoc_co2_npp_global_monthly_1971_2012.nc4
LPJmL	WATCH-WFDEI	lpjml_watch-wfdei_nobc_hist_varsoc_co2_npp_global_monthly_1971_2010.nc4
ORCHIDEE	GSWP3	orchidee_gswp3_nobc_hist_varsoc_co2_npp_global_monthly_1971_2010.nc4
ORCHIDEE	PGMFD v2.1	orchidee_princeton_nobc_hist_varsoc_co2_npp_global_monthly_1971_2012.nc4
ORCHIDEE	WATCH-WFDEI	orchidee_watch-wfdei_nobc_hist_varsoc_co2_npp_global_monthly_1971_2010.nc4

To assess uncertainty related to these 12 models, we first disaggregated and resampled the data to 5 arc-min resolution and masked it to our grassland areas. Then, based on the range of 12 values we had for each cell, we created a truncated normal distribution (n = 1000) for each cell and each year. The factors limiting AGB (temperature, TreeCoverMultiplier, SlopesMultiplier) were then applied to the distributions similarly as explained in S2.4-S2.7. For this uncertainty comparison, we calculated coefficients of variation (CV), related to ISIMIP derived AGB, for each cell.

We also derived interannual variability (IV) from the Monte Carlo simulated data (n = 1000) and compared the MODIS NPP based AGB to ISIMIP based AGB. The results are presented in Figure S9. Please note that instead of the time period from 2001 to 2015 used elsewhere in this study, we calculated interannual variability for 2001-2010, as those years were compatible with the MODIS NPP based analysis.

S3 Other supplementary figures

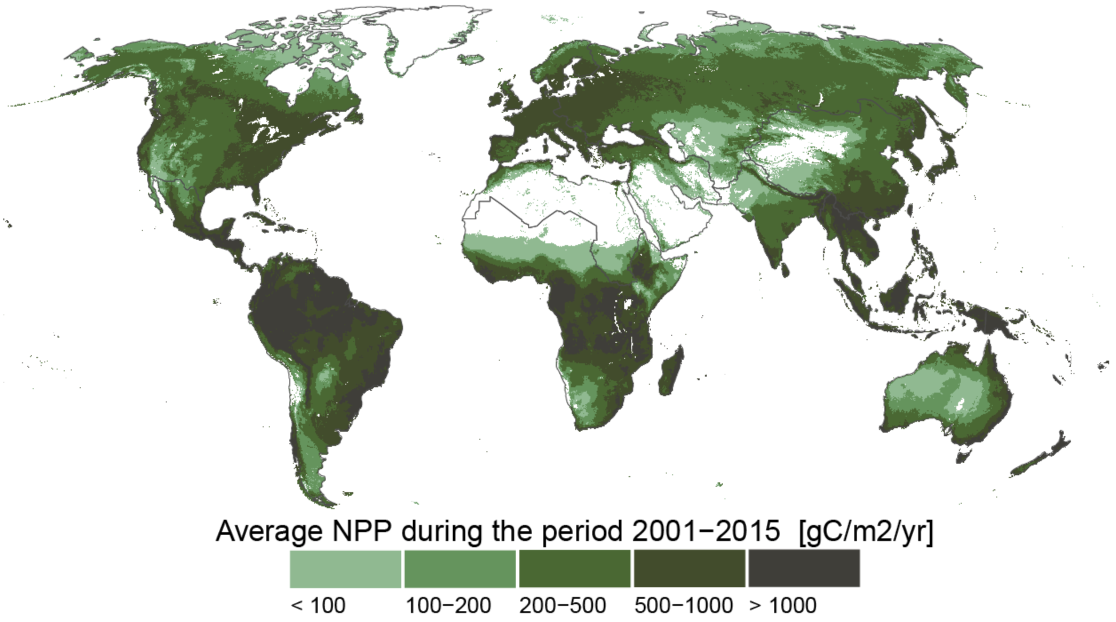

Figure S2. Average Net Primary Productivity (NPP) over 2001-2015. Derived from MODIS; see Section 2.1 in the main article. Source: Running and Zhao (2019)

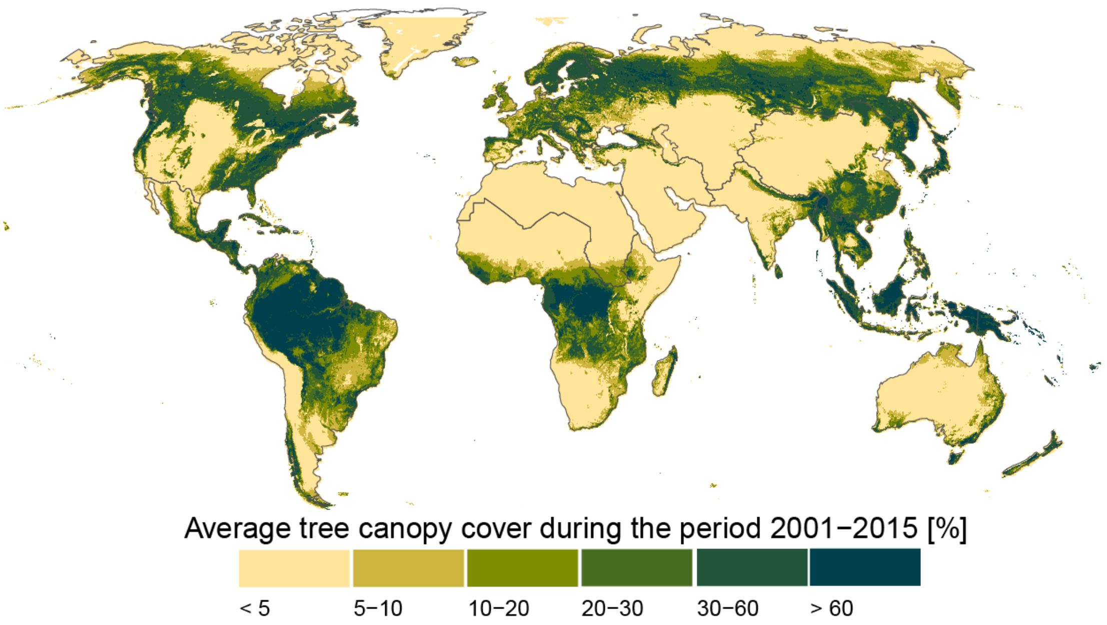

Figure S3. Average tree canopy cover (in %) over 2001-2015. Source: Sexton et al. (2013).

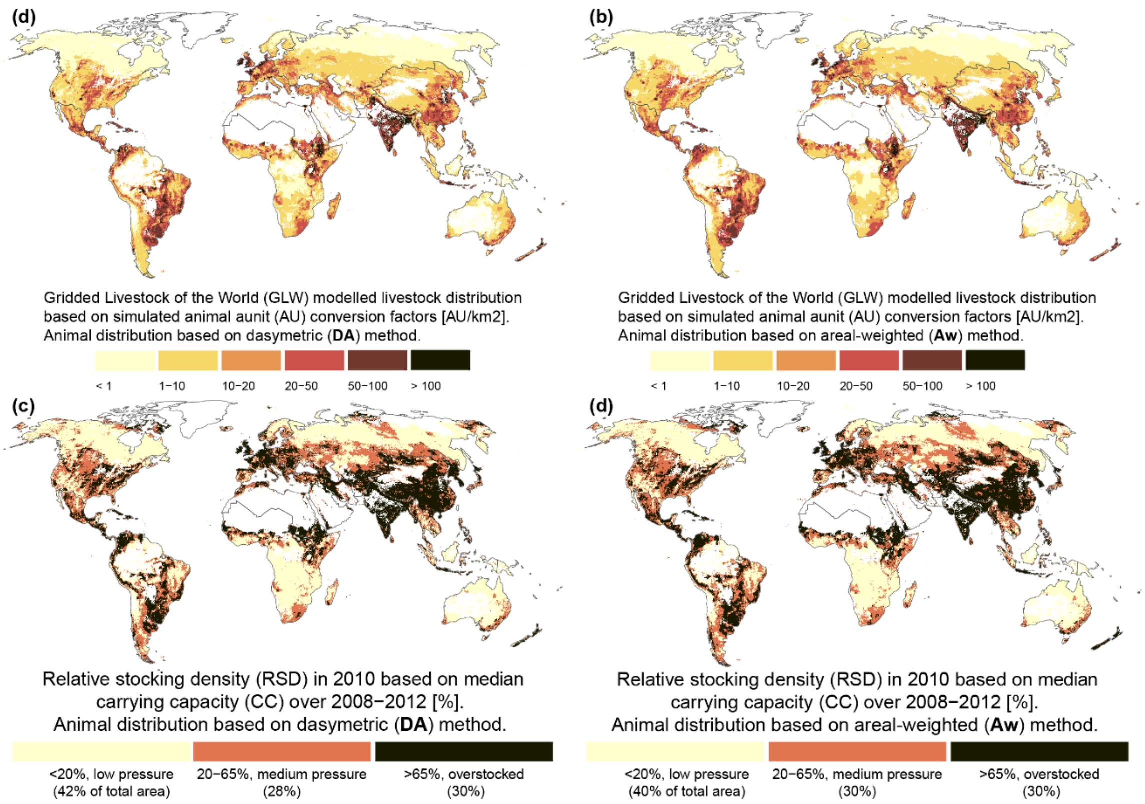

Figure S4. Gridded Livestock of the World (GLW) modelled livestock distribution in 2010 based on simulated data (a, b) (see different animal unit (AU) conversion factors in S2.8) and the relative stocking density (RSD) (c,d), i.e. the ratio of carrying capacity (CC) used by the Gridded Livestock of the World (GLW) modelled livestock (industrial and grazing) on the world's grasslands in 2010 based on the median CC of 2008-2012 (for each year, 2008-2012, we calculated RSD by dividing simulated livestock estimates by simulated CC for that year. Then we selected the median of those five annual values). Tiles (a) and (c) calculated using dasymetric (DA) method of GLW. Tiles (b) and (d) calculated using areal-weighted (Aw) method of GLW. Source: Gridded Livestock of the World (Gilbert et al., 2018).

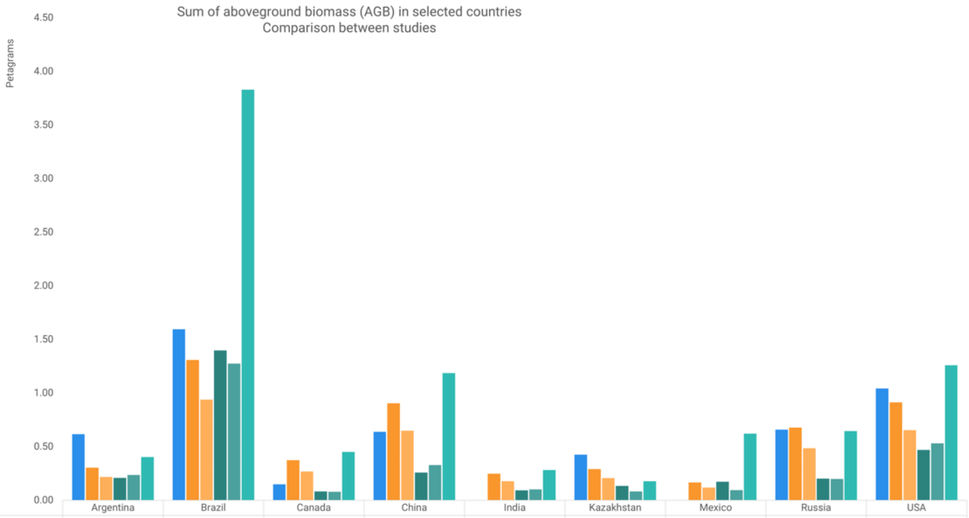

	Argentina	Brazil	Canada	China	India	Kazakhstan	Mexico	Russia	USA
Sun et al. (2021)	0.62	1.60	0.15	0.64		0.43		0.66	1.04
Wolf et al. (2021): AGB = 0.60 * NPP	0.30	1.31	0.37	0.90	0.25	0.29	0.17	0.68	0.91
Wolf et al. (2021): AGB = 0.43 * NPP	0.22	0.94	0.27	0.65	0.18	0.21	0.12	0.49	0.65
Present study, MODIS NPP based AGB with restrictions	0.21	1.40	0.08	0.26	0.09	0.13	0.17	0.20	0.47
Present study, ISIMIP2a based AGB with restrictions	0.24	1.27	0.08	0.33	0.10	0.08	0.10	0.20	0.53
Present study, MODIS NPP based AGB without restrictions	0.40	3.83	0.45	1.19	0.28	0.18	0.62	0.65	1.26

Figure S5. Total aboveground biomass (AGB) estimates in selected countries. Comparison between this study, Wolf et al. (2021) and Sun et al. (2021). In the present study, NPP partitioning was implemented using temperature as a predictor (see Methods), whereas Wolf et al. (2021) used a single constant (0.60 or 0.43) to derive aboveground fraction from the NPP. The work of Sun et al (2021) is based on many field measurements and random forest model. Total AGB estimates of ours and Wolf et al. (2021) are based on median AGB values over 2006-2010, whereas the timespan is much longer in the study of Sun et al. (2021). For this comparison, we estimated total AGB also using ISIMIP2a modelled NPP data as input. Moreover, we also estimated AGB using MODIS NPP, but without any restrictions (see Methods).

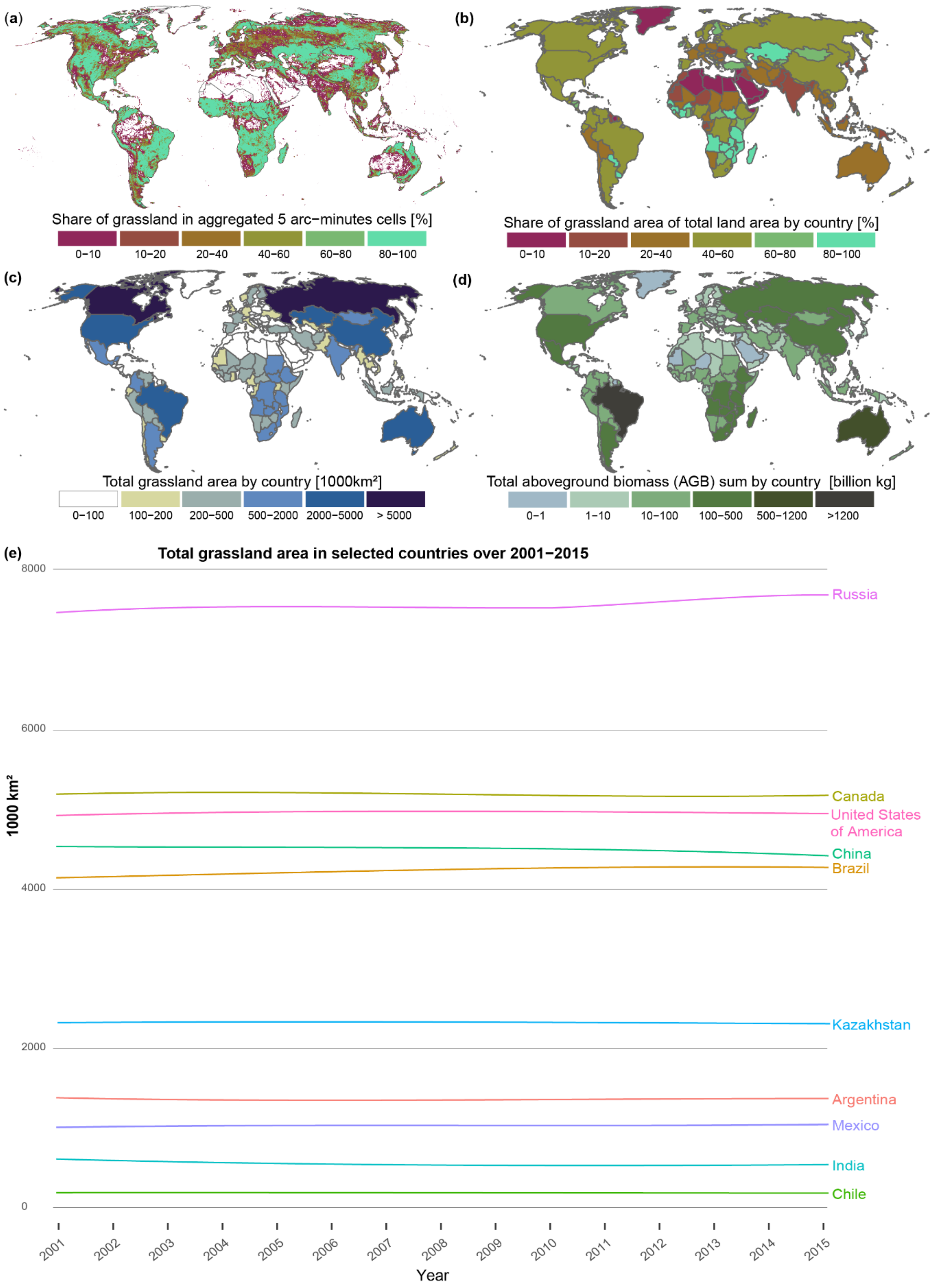

Figure S6. Share of grassland area in aggregated 5arc-minutes cells (a), the share of grassland area of the total land area of the country (b), total grassland area by country (c), total AGB by country based on median AGB values over 2008-2012 (d), and total grassland area in selected countries over 2001-2015. Tiles (a-d) are based on the mode value of land cover type over 2001-2015, whereas in tile (d) we used different land cover type for each year. Tabulated data for tiles (b-e) is available in the supplementary (Sheet S2; Sheet S3). See the data availability statement.

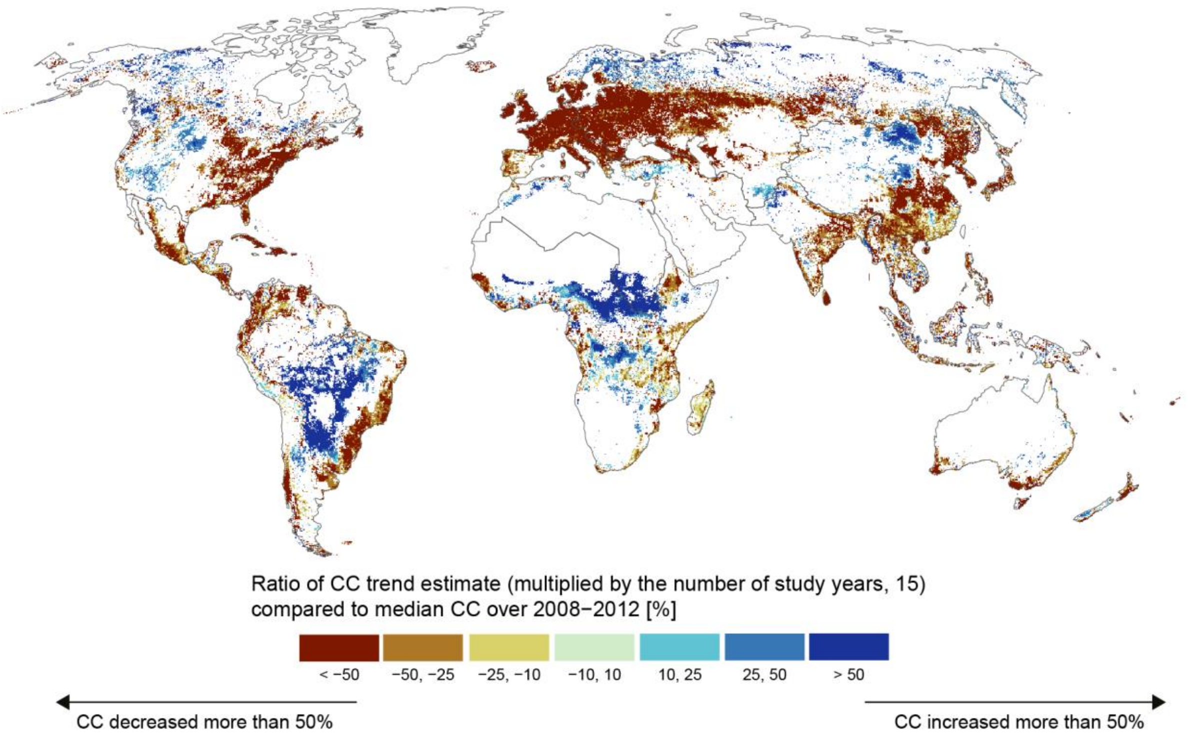

Figure S7. The ratio of carrying capacity (CC) trend estimate, multiplied by the number of study years (15) compared to median CC over 2008-2012. Masked to areas where the trend is significant (see Figure 2b).

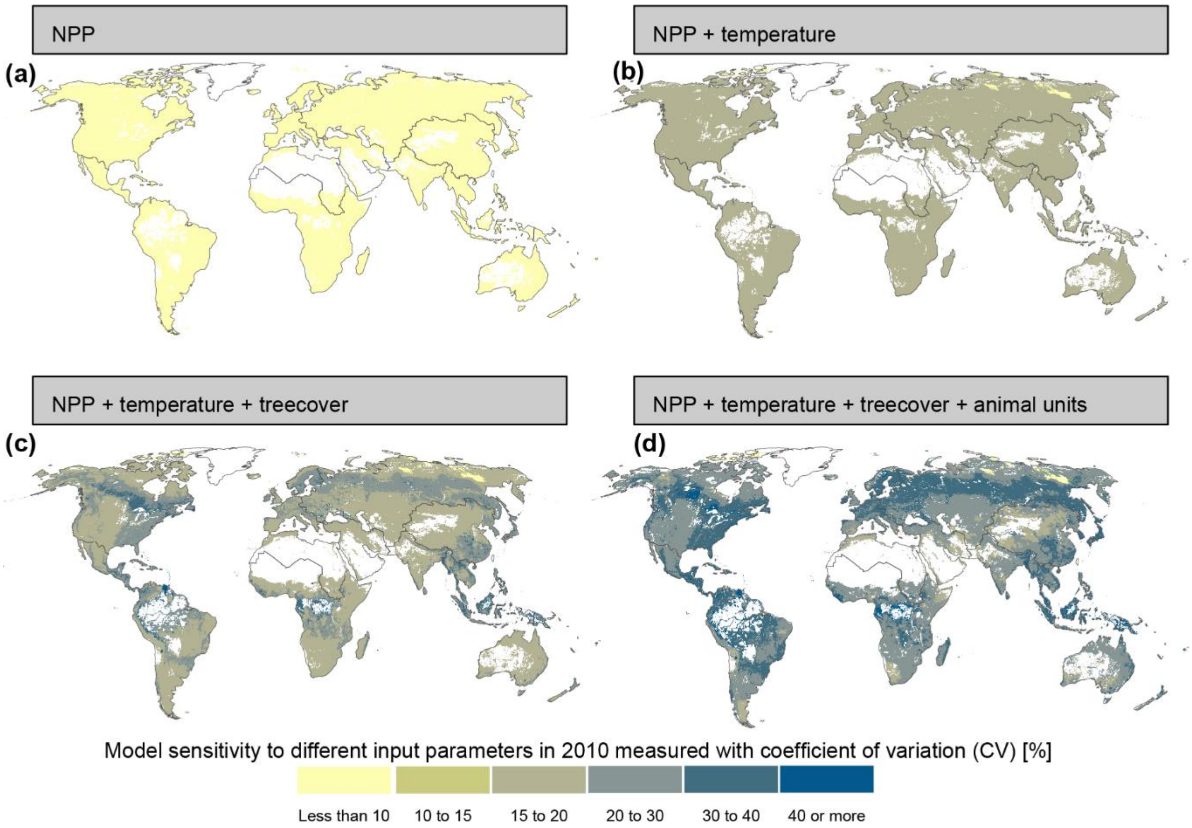

Figure S8. Model sensitivity to different input parameters in 2010, measured with the coefficient of variation (CV). Tile a) only the variable net primary productivity (NPP) included in the simulation, b) variables NPP and annual temperature included c) variables NPP, annual temperature and tree cover included, d) variables NPP, annual temperature, tree cover and animal unit conversion factors included. All the maps presented here are based on yearly median values of Monte Carlo simulated data (n = 1000) as presented in methods and in the appendix and masked to areas where AGB is larger than 0.1 g m-2 yr-1. Note that too cold regions (colder than -13C), that yield negative AGB estimates are not removed from this figure.

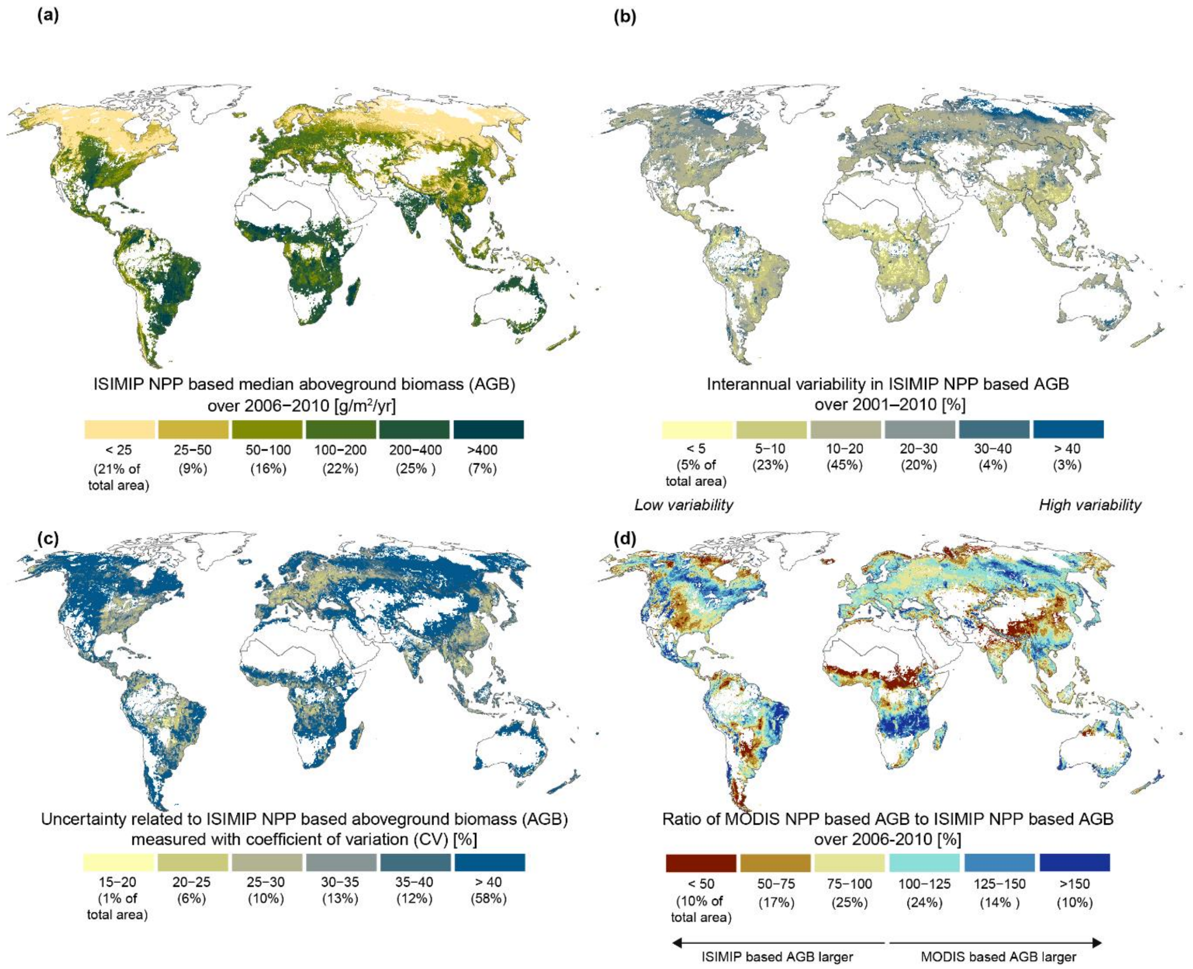

Figure S9. (a): ISIMIP2a model ensemble NPP based median aboveground biomass (AGB) over 2006-2010 (first, we calculated median simulated AGB for each year and then selected the median of those five values); (b): interannual variability of ISIMIP2a NPP based AGB over 2001-2010; (c): uncertainty of ISIMIP2a NPP based AGB) originating from uncertainties in input data, measured with the coefficient of variation (CV). First, we calculated the CV for each year and then selected the median of those five CV values. The CVs for individual years were calculated from Monte Carlo runs (n = 1000); (d): ratio of MODIS NPP based AGB estimates to ISIMIP2a NPP based AGB estimates over 2006-2010. Calculated as MODIS NPP based AGB divided by ISIMIP2a NPP based AGB.

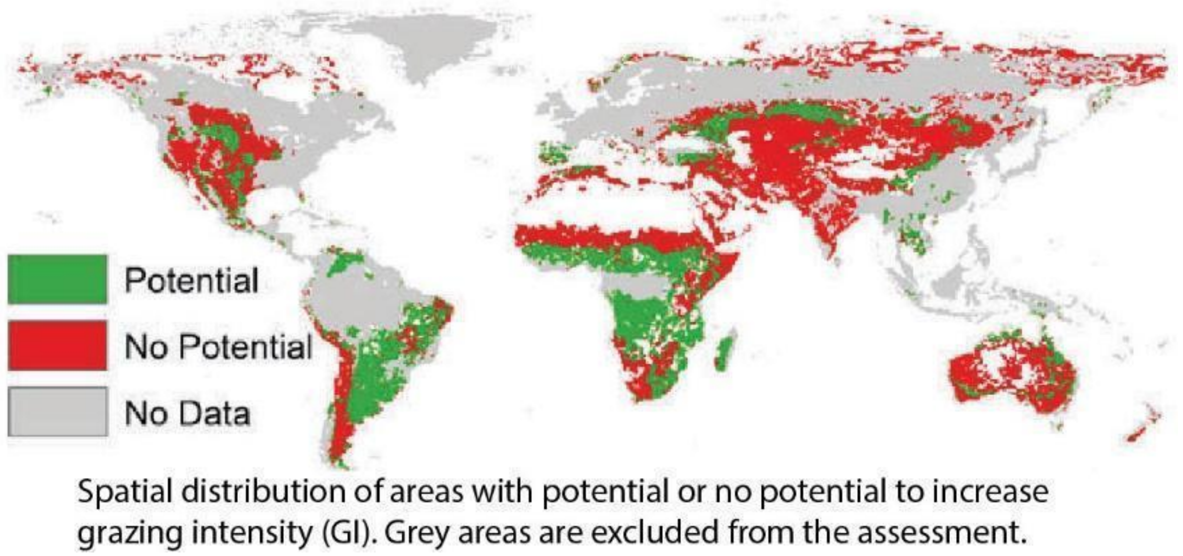

Figure S10. Spatial distribution of areas with potential or no potential to increase grazing intensity (GI). Grey areas are excluded from the assessment (Fetzel et al., 2017). Reproduced by permission, © 2017 John Wiley & Sons Ltd, Global Change Biology, 23, 1636–1647.

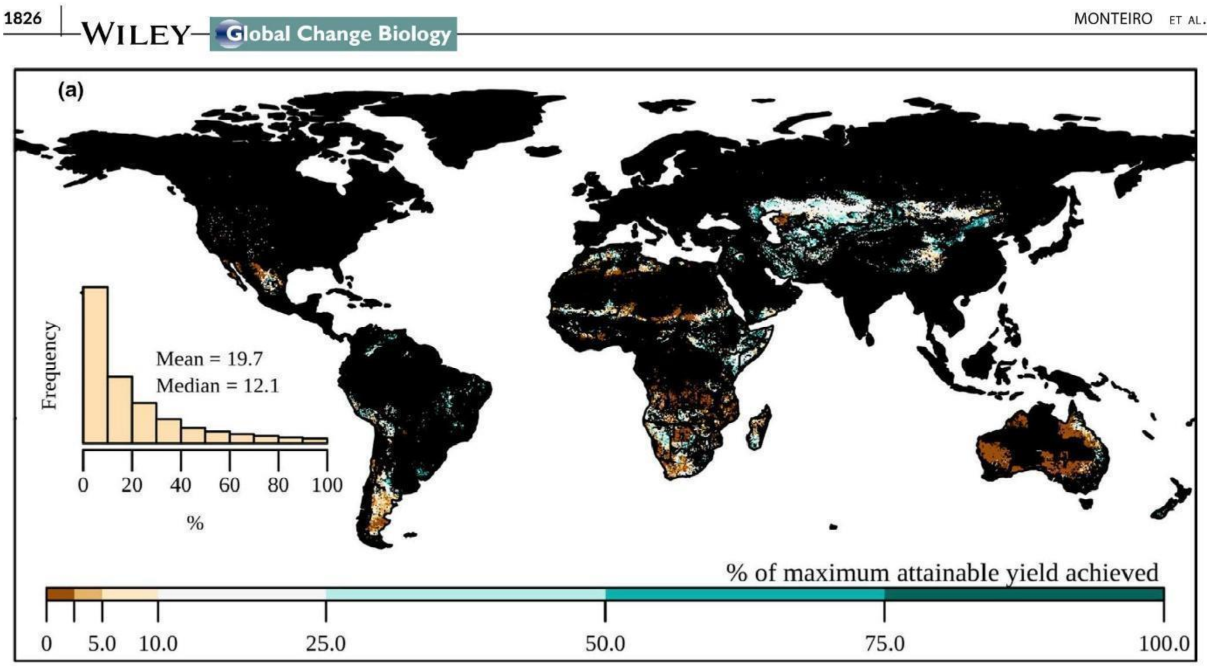

Figure S11. Yield gaps for all pasture lands (5′ × 5′ spatial resolution), considering total protein from meat and milk produced by cattle, sheep, or goats. (a) Yield gap expressed as the percent of achieved yield relative to the climate-adjusted maximum (Y95) (Monteiro et al., 2020). Reproduced by permission © 2020 John Wiley & Sons Ltd, Global Change Biology, 26, 1820–1832.

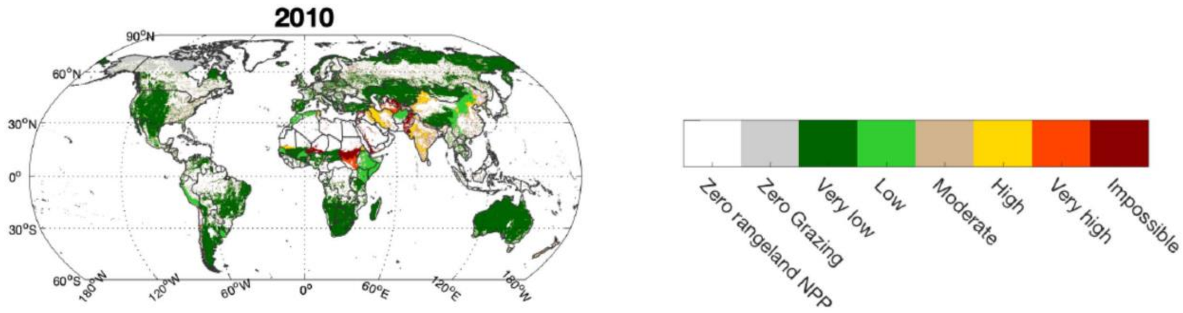

Figure S12. Grazing intensity (GI) (percent of aboveground net primary productivity ANPP removed by grazing) by nation or state in 2010 (for Argentina, Brazil, Canada, Chile, China, India, Kazakhstan, Mexico, Russian Federation, and United States). Colors|categories represent the following ranges of GI: dark green|very low: 0 > GI 20%; light green|low: 20 > GI 40%; tan|moderate: 40 > GI 60%; yellow|high: 60 > GI 80%; orange |very high: 80 > GI 100%; dark red | impossible: GI 100% of ANPP. Source: (Wolf et al., 2021; excerpt from Figure 1).

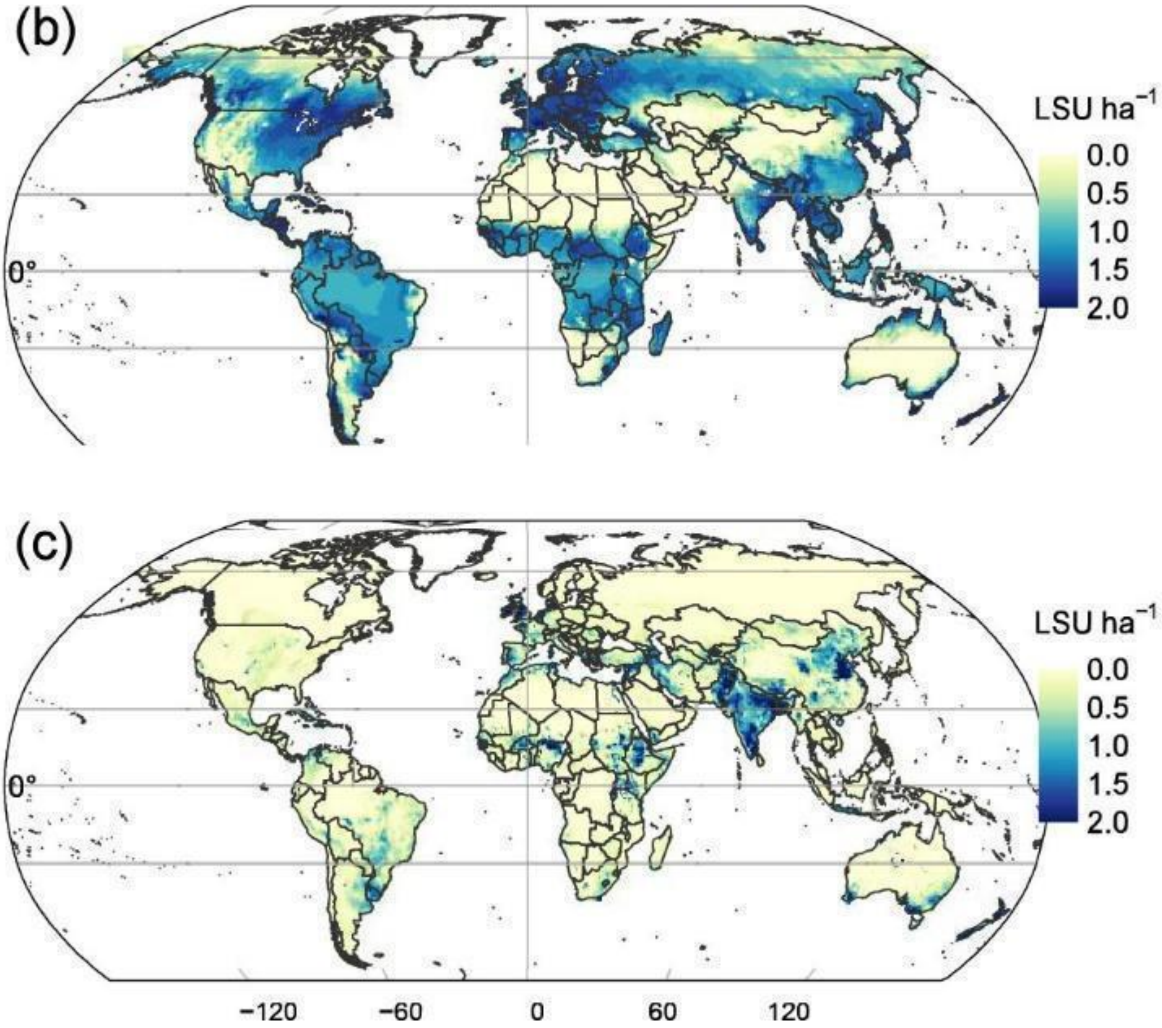

Figure S13. “Distribution of livestock densities that result in (b) maximum LSU (Livestock Unit) that can be continuously supported by grazing only (LSUfeed in LSU ha-1) under harvest option GD averaged over the years 1998 to 2002. Reported livestock densities in pastoral and mixed livestock production systems (Robinson et al., 2014) are given as a comparison in panel (c) (Rolinski et al., 2018)“. This figure is licensed under the Creative Commons Attribution 4.0 License.

S4 References

Beer, C., Reichstein, M., Tomelleri, E., Ciais, P., Jung, M., Carvalhais, N., Rödenbeck, C., Arain, M.A., Baldocchi, D., Bonan, G.B., Bondeau, A., Cescatti, A., Lasslop, G., Lindroth, A., Lomas, M., Luyssaert, S., Margolis, H., Oleson, K.W., Roupsard, O., Veenendaal, E., Viovy, N., Williams, C., Woodward, F.I., Papale, D., 2010. Terrestrial Gross Carbon Dioxide Uptake: Global Distribution and Covariation with Climate. Science 329, 834–838. https://doi.org/10.1126/science.1184984

De Leeuw, J., Rizayeva, A., Namazov, E., Bayramov, E., Marshall, M.T., Etzold, J., Neudert, R., 2019. Application of the MODIS MOD 17 Net Primary Production product in grassland carrying capacity assessment. International Journal of Applied Earth Observation and Geoinformation 78, 66–76. https://doi.org/10.1016/j.jag.2018.09.014

De Leeuw, P.N., Tothill, J.C., 1990. The concept of rangeland carrying capacity in sub-Saharan Africa: Myth or reality. Overseas Development Institute, Pastoral Development Network London.

Eggleston, S., Buendia, L., Miwa, K., Ngara, T., Tanabe, K., 2006. 2006 IPCC guidelines for national greenhouse gas inventories. Institute for Global Environmental Strategies Hayama, Japan.

Fetzel, T., Havlik, P., Herrero, M., Erb, K.-H., 2017. Seasonality constraints to livestock grazing intensity. Global Change Biology 23, 1636–1647. https://doi.org/10.1111/gcb.13591

George, M., Lyle, D., 2009. Stocking Rate and Carrying Capacity. Module 4: Ranch Operations and Grazing Management. Ecology and Management of Grazing, an Online Course.

Gilbert, M., Nicolas, G., Cinardi, G., Van Boeckel, T.P., Vanwambeke, S.O., Wint, G.R.W., Robinson, T.P., 2018. Global distribution data for cattle, buffaloes, horses, sheep, goats, pigs, chickens and ducks in 2010. Scientific Data 5, 180227. https://doi.org/10.1038/sdata.2018.227

Holechek, J., Pieper, R.D., Herbel, C.H., 2010. Range management : principles and practices, Sixth edition. ed. Prentice Hall, Upper Saddle River, N.J. : London.

Hui, D., Jackson, R.B., 2006. Geographical and interannual variability in biomass partitioning in grassland ecosystems: a synthesis of field data. New Phytologist 169, 85–93. https://doi.org/10.1111/j.1469-8137.2005.01569.x

Keuper, F., Wild, B., Kummu, M., Beer, C., Blume-Werry, G., Fontaine, S., Gavazov, K., Gentsch, N., Guggenberger, G., Hugelius, G., Jalava, M., Koven, C., Krab, E.J., Kuhry, P., Monteux, S., Richter, A., Shahzad, T., Weedon, J.T., Dorrepaal, E., 2020. Carbon loss from northern circumpolar permafrost soils amplified by rhizosphere priming. Nature Geoscience 13, 560–565. https://doi.org/10.1038/s41561-020-0607-0

Le Brocque, A.F., Goodhew, K.A., Cockfield, G., 2008. Retaining trees in a grazing landscape: impacts on ground cover in sheep-grazing agro-ecosystems in southern Queensland, in: Veg Futures 08 Conference Proceedings. Greening Australia, p. 30.

Lloyd, J., Bird, M.I., Vellen, L., Miranda, A.C., Veenendaal, E.M., Djagbletey, G., Miranda, H.S., Cook, G., Farquhar, G.D., 2008. Contributions of woody and herbaceous vegetation to tropical savanna ecosystem productivity: a quasi-global estimate. Tree physiology 28, 451–468. https://doi.org/10.1093/treephys/28.3.451

Monteiro, L.A., Allee, A.M., Campbell, E.E., Lynd, L.R., Soares, J.R., Jaiswal, D., Oliveira, J. de C., Vianna, M. dos S., Morishige, A.E., Figueiredo, G.K.D.A., Lamparelli, R.A.C., Mueller, N.D., Gerber, J., Cortez, L.A.B., Sheehan, J.J., 2020. Assessment of yield gaps on global grazed-only permanent pasture using climate binning. Global Change Biology 26, 1820–1832. https://doi.org/10.1111/gcb.14925

Paul, S.S., Mandal, A.B., Kannan, A., Mandal, G.P., Pathak, N.N., 2003. Comparative dry matter intake and nutrient utilisation efficiency in lactating cattle and buffaloes. Journal of the Science of Food and Agriculture 83, 258–267.

Petz, K., Alkemade, R., Bakkenes, M., Schulp, C.J.E., van der Velde, M., Leemans, R., 2014. Mapping and modelling trade-offs and synergies between grazing intensity and ecosystem services in rangelands using global-scale datasets and models. Global Environmental Change 29, 223–234. https://doi.org/10.1016/j.gloenvcha.2014.08.007

Priyadarshini, K.V.R., Prins, H.H.T., Bie, S. de, Heitkönig, I.M.A., 2016. The facilitative role of trees in tree-grass interactions in savannas.

Reyer, C., Asrar, G., Betts, R., Chang, J., Chen, M., Ciais, P., Dury, M., Francois, L., Henrot, A., Hickler, T., Ito, A., Jacquemin, I., Nishina, K., Mishurov, M., Morfopoulos, C., Munhoven, G., Ostberg, S., Pan, S., Rafique, R., Schaphoff, S., Steinkamp, J., Tian, H., Ren, W., Yang, J., Zeng, N., Zhao, F., Büchner, M., 2019. ISIMIP2a Simulation Data from Biomes Sector (V. 1.1). https://doi.org/10.5880/PIK.2019.005

Rolinski, S., Müller, C., Heinke, J., Weindl, I., Biewald, A., Bodirsky, B.L., Bondeau, A., Boons-Prins, E., Bouwman, A., Leffelaar, P., Roller, J.T., Schaphoff, S., Thonicke, K., 2018. Modeling vegetation and carbon dynamics of managed grasslands at the global scale with LPJmL 3.6. Geosci. Model Dev. 24. https://hal-amu.archives-ouvertes.fr/hal-01788178

Running, S.W., Zhao, M., 2019. MOD17A3HGF MODIS/Terra Net Primary Production Gap-Filled Yearly L4 Global 500 m SIN Grid V006. 2019, distributed by NASA EOSDIS Land Processes DAAC 35. https://doi.org/10.5067/MODIS/MOD17A3HGF.006

Saatchi, S.S., Harris, N.L., Brown, S., Lefsky, M., Mitchard, E.T.A., Salas, W., Zutta, B.R., Buermann, W., Lewis, S.L., Hagen, S., Petrova, S., White, L., Silman, M., Morel, A., 2011. Benchmark map of forest carbon stocks in tropical regions across three continents. PNAS 108, 9899–9904. https://doi.org/10.1073/pnas.1019576108

Sexton, J.O., Song, X.-P., Feng, M., Noojipady, P., Anand, A., Huang, C., Kim, D.-H., Collins, K.M., Channan, S., DiMiceli, C., Townshend, J.R., 2013. Global, 30-m resolution continuous fields of tree cover: Landsat-based rescaling of MODIS vegetation continuous fields with lidar-based estimates of error. International Journal of Digital Earth 6, 427–448. https://doi.org/10.1080/17538947.2013.786146

Singhal, K.K., Mohini, M., Jha, A.K., Gupta, P.K., 2005. Methane emission estimates from enteric fermentation in Indian livestock: Dry matter intake approach. Current Science 119–127.

Sulla-Menashe, D., Friedl, M.A., 2018. User guide to collection 6 MODIS land cover (MCD12Q1 and MCD12C1) product. USGS: Reston, VA, USA 1–18. https://doi.org/10.5067/MODIS/MCD12Q1.006

Wolf, J., Chen, M., Asrar, G.R., 2021. Global Rangeland Primary Production and Its Consumption by Livestock in 2000–2010. Remote Sensing 13, 3430. https://doi.org/10.3390/rs13173430
